# Visible Light-Induced Aerobic Oxidative Dehydrogenation of C–N/C–O to C=N/C=O Bonds Using Metal-Free Photocatalysts: Recent Developments

**DOI:** 10.3390/molecules27020497

**Published:** 2022-01-13

**Authors:** Alejandro Torregrosa-Chinillach, Rafael Chinchilla

**Affiliations:** Department of Organic Chemistry, Faculty of Sciences, Institute of Organic Synthesis (ISO), University of Alicante, Apdo. 99, 03080 Alicante, Spain; alex.torregrosa@ua.es

**Keywords:** oxidative dehydrogenation, photocatalysis, metal-free, C–N bond, C–O bond

## Abstract

Performing synthetic transformation using visible light as energy source, in the presence of a photocatalyst as a promoter, is currently of high interest, and oxidation reactions carried out under these conditions using oxygen as the final oxidant are particularly convenient from an environmental point of view. This review summarizes the recent developments achieved in the oxidative dehydrogenation of C–N and C–O bonds, leading to C=N and C=O bonds, respectively, using air or pure oxygen as oxidant and metal-free homogeneous or recyclable heterogeneous photocatalysts under visible light irradiation.

## 1. Introduction

Photocatalysis has become one of the most powerful and efficient strategies employed in organic synthesis in recent years, emerging as a sustainable and environmentally friendly alternative methodology in electron transfer-mediated chemical transformations [1,2,3,4,5]. This fact is reflected in the impressive and rapid increase in the number of publications in this field in the last decade. In this context, the use of visible light is singularly attractive due to its renewability and low cost and because organic compounds do not absorb it [6,7,8,9]. Therefore, possible side reactions produced in traditional photochemical transformations using UV light, which is absorbed directly by organic molecules, are avoided with the employment of innocuous and less energetic visible light, its use also simplifying the reaction setup. The use of visible light implies the need of using substoichiometric amounts of a photocatalyst [10].

Among all the photocatalysts, transition metal-based compounds [11,12,13,14], metal-free molecules [3,15,16,17,18,19] and semiconductors [20,21] are the most utilized. These promoters have been applied successfully to many photochemical transformations, such as oxidations [22,23,24,25,26], cycloadditions [27,28,29,30], C–H bond functionalizations [31,32,33,34], photoredox reactions [6,16,17,35,36,37,38,39,40,41,42,43] and even enantioselective reactions [44,45,46,47,48,49,50,51]. In addition, other effective photocatalysts have been widely applied for photocatalysis in energy and environment [52,53,54].

The oxidation of organic compounds is one of the most performed transformations in organic synthesis, since it is extensively used in the industry to synthesize drugs and fine chemicals [55,56]. Traditional oxidation methodologies use stoichiometric amounts of manganese-, chromium- or hypervalent halogen-based oxidants, which are toxic, have low selectivities, are expensive and generate undesired waste, as well as catalysts based on noble metals. This fact has become unacceptable from an environmental point of view, with the development of new greener and sustainable strategies for carrying out oxidation reactions employing molecular oxygen as an economic and benign oxidant gaining interest [57].

Thus, molecular oxygen present as pure O_2_ or in atmospheric air is the cheapest, greenest and most readily available oxidant that can be used in oxidative transformations. The combination of O_2_ with a catalytic system makes the oxidation strategy very attractive for developing environmentally friendly and sustainable protocols, although it is a challenge in organic chemistry [58,59]. Molecular oxygen needs to be activated to generate reactive oxygen species (ROS), photocatalysis then emerging as an attractive way to achieve oxidation reactions [60].

Visible light-induced oxidations for the synthesis of oxygen-containing organic molecules have become an interesting and promising area of research [61]. In particular, the basic selective aerobic oxidative dehydrogenation of alcohols to the corresponding carbonyl compounds is a well-known and very important reaction in the industry [62]. In addition, imines are relevant nitrogen-containing sources in drugs and organic synthesis, the aerobic oxidative dehydrogenation of amines being a sustainable and environmentally friendly strategy to synthesize them [24,25,61].

Several reviews about visible light-induced oxidations have been published up to now, although they are mainly centered on a specific photocatalyst and in metal-catalyzed homogenous or heterogeneous photo-oxidations [7,20,24,25,26,61,62,63,64,65]. In this review, we summarize the developments in the metal-free photocatalytic aerobic oxidative dehydrogenation of C–N and C–O bonds to achieve C=N and C=O bonds under homogeneous and heterogeneous conditions in the last six years.

## 2. Oxidative Dehydrogenation of C–N Bonds

### 2.1. Homogeneous Oxidative Dehydrogenation of C–N Bonds

**Scheme 1:**
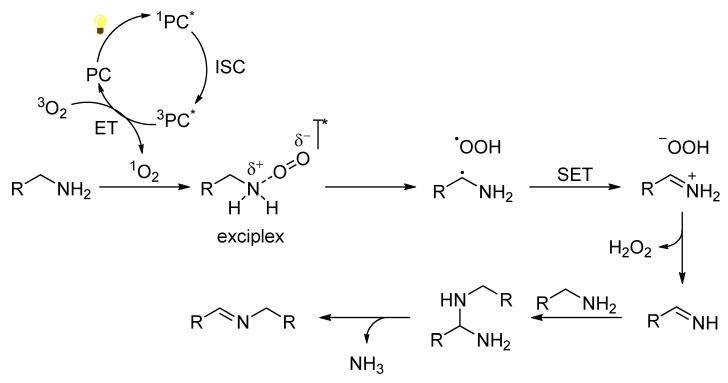
Commonly accepted mechanism of the oxidative dehydrogenation of amines to imines and subsequent homocoupling, mediated by singlet oxygen.

The homogeneous oxidative dehydrogenation of amines by air or pure O_2_ is a very interesting strategy for the preparation of imines. The commonly accepted mechanism of this transformation [25,66,67,68] begins with the formation of the singlet excited state of the photocatalyst (^1^PC*) by irradiation with visible light (Scheme 1). Next, the triplet excited state (^3^PC*) is produced by intersystem crossing (ISC), and the singlet oxygen (^1^O_2_) is generated by energy transfer (ET) from the triplet oxygen (^3^O_2_) [69]. At this point, the starting amine forms an exciplex with the singlet oxygen as an intermediate on which a hydrogen abstraction and a single electron transfer (SET) process occur. After that, hydrogen peroxide is generated leading to an imine. This species can also react with the starting amine to obtain an homocoupled imine and ammonia as a by-product.

**Scheme 2:**
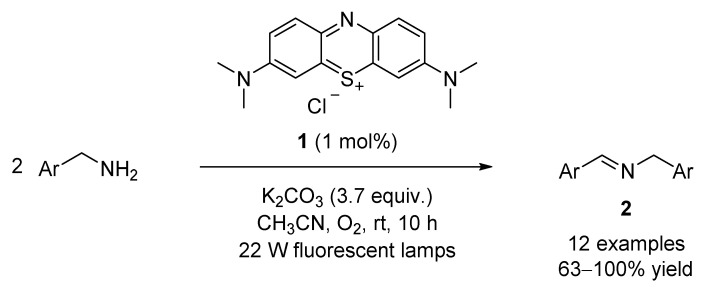
Aerobic photo-oxidative coupling of benzylamines to render *N*-benzylidene benzylamines **2** catalyzed by **1**.

Based on this transformation, several methodologies have been carried out in recent years. Thus, methylene blue (**1**), an organic dye, was employed as photocatalyst in the photo-organocatalytic aerobic oxidative coupling of benzylamines to obtain *N*-benzylidene benzylamines **2** in good yields (Scheme 2) [70].

The methodology in Scheme 2 shows the photocatalytic aerobic oxidative coupling of the same primary amine, raising doubt as to which side the oxidation to the imine occurs on if this coupling is carried out between two different primary amines. To solve this question, a theoretical study on what factors influence the oxidation side in the aerobic photo-oxidative coupling of two different primary amines was performed [71]. Thus, tetraphenylporphyrin (TPP) was employed as photocatalyst in the oxidation of non-symmetric aromatic secondary amines, suggesting that the oxidation side depends directly on the bond dissociation energy (BDE) of the C–H bond next to the nitrogen atom. To quantify the selectivity, a theoretically natural bond order analysis (NBO) and an experimental analysis of the ^1^*J*_CH_ coupling constants obtained by NMR spectroscopy, both expressed in terms of hybridization or %s character, were performed, showing a strong inductive effect on hybridization. This means that electronegative groups at the ortho position cause the benzylic C–H bond to have less p character, increasing the %s character and the regioselectivity to one oxidation side. In addition, one of the aromatic rings was exchanged with an alkyl group with less p character to find out if steric effects influence the oxidation side. Thus, when secondary amines bearing neopentyl residues were submitted to the same oxidation strategy, an inversion in selectivity was observed, favoring the oxidation of the alkyl side.

Apart from the example shown in Scheme 2, other methodologies have been applied in the same transformation. Thus, TPP and methylene blue **1** were used in the aerobic oxidative coupling of benzylamine to *N*-benzylidene benzylamine using a gas-liquid membrane reactor under homogeneous continuous flow at 90 and 100 °C, respectively, and under white light irradiation, obtaining moderate yields [72]. This strategy is interesting because it enables a safe and accurate mass transport of oxygen, adjusting the gas pressure, the temperature of the membrane and the liquid flow rate, making it possible to maintain reproducible conditions with better control of the process.

**Figure 1:**
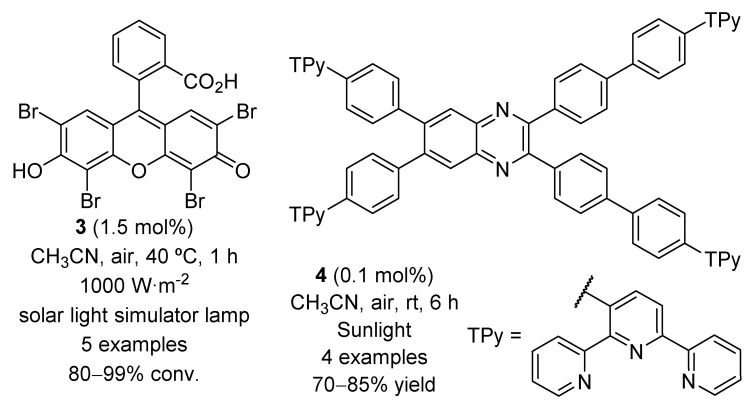
Results obtained in the aerobic photo-oxidative coupling of benzylamines to render *N*-benzylidene benzylamines **2** catalyzed by **3** and **4**.

Sunlight is a sustainable alternative source of energy that can be employed in photocatalysis. Thus, the aerobic oxidative coupling of benzylamines to render **2** under sunlight irradiation has been carried out using very low loadings of the xanthene-based photocatalyst Eosin Y (**3**) [73] and a terpyridine-based supramolecular nanoassembly of pyrazine-based donor–acceptor systems, (TETPY, **4**) [74], both catalysts showing excellent activity (Figure 1). In the first case, the fact that it could be possible to mimic the solar light environment with a sunlight simulator lamp makes the system very attractive, while in the second case carried out under direct real sunlight irradiation, the nanoassemblies have excellent aggregation-induced emission enhancement (AIEE) and intramolecular charge transfer (ICT), characteristics added to their potential to generate ROS.

**Scheme 3:**
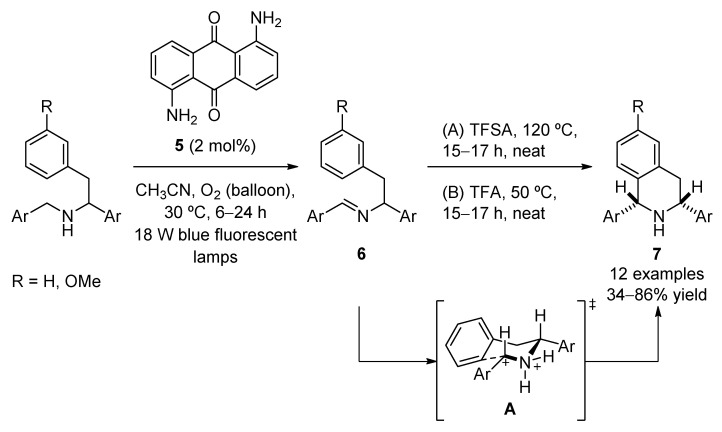
Photo-organocatalytic synthesis of *cis*-1,3-diaryl tetrahydroisoquinolines **7** by aerobic oxidative amine dehydrogenation/super acid-mediated Pictet–Spengler cyclization.

Interestingly, an anthraquinone-based photocatalyst (**5**), which previously showed excellent activity in the oxidative coupling of benzylamines to *N*-benzylidene benzylamines **2** [75], has also been used to synthesize *cis*-1,3-diaryl tetrahydroisoquinolines **7** through a photo-organocatalytic aerobic oxidative amine dehydrogenation/super acid-mediated Pictet–Spengler cyclization (Scheme 3) [75]. Thus, α-benzyl dibenzyl-amines were regioselectively converted into the corresponding branched aldimines **6** by aerobic oxidative dehydrogenation under visible light (Scheme 3). Without further purification, these aldimines were submitted to an acid-mediated cyclization using trifluoromethanesulfonic acid (TFSA) or trifluoroacetic acid (TFA), leading to the respective tetrahydroisoquinolines **7** in moderate to good yields. The diastereoselectivity of **7** could be explained with the chair-like transition state **A**, in which the aryl groups are in a pseudo equatorial position.

**Scheme 4:**
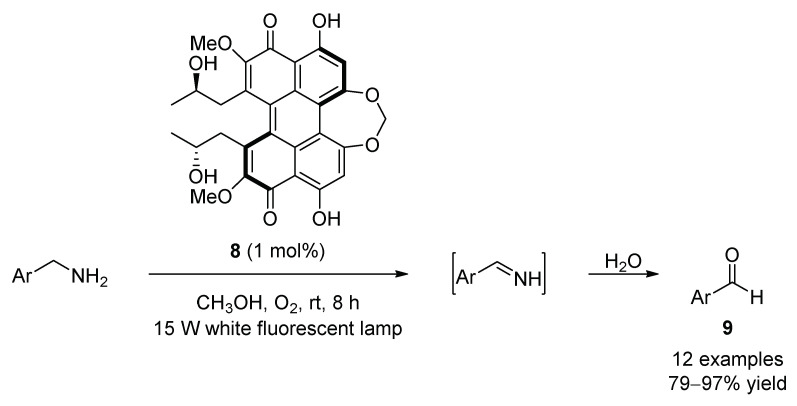
Aerobic photo-oxidation of amines to aldehydes **9** catalyzed by **8**.

Imines obtained from the photo-oxidation of amines can also be transformed into aldehydes due to the presence of H_2_O in the reaction media. Thus, a cercosporin-bioinspired photocatalyst **8**, which is a perylenequinonoid pigment, has been obtained from microbial fermentation of glucose and employed in the oxidation of amines to aldehydes **9** under irradiation with white light, obtaining good to excellent yields (Scheme 4) [76].

**Scheme 5:**
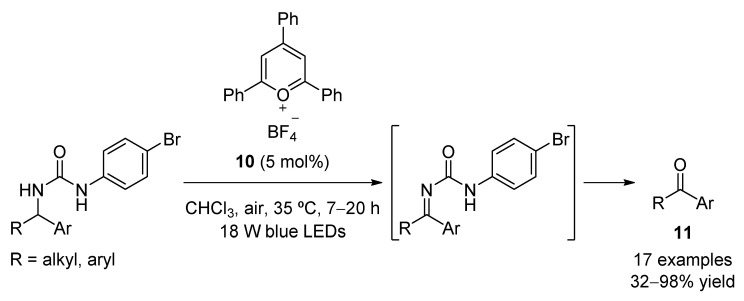
Aerobic photo-oxidative dehydrogenation of urea-protected benzylamines leading to ketones **11** catalyzed by **10**.

The oxidative dehydrogenation of benzylamines protected with a urea moiety was explored, employing 2,4,6-triphenylpyrylium tetrafluoroborate (**10**) as photocatalyst, leading to the corresponding ketones **11** in moderate to excellent yields (Scheme 5) [77]. The interest in using urea-protected benzylamines resides in avoiding the possible oxidative coupling of the benzylamines.

**Scheme 6:**
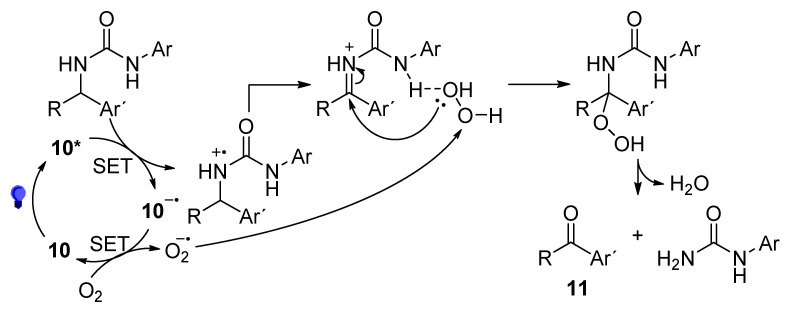
Proposed mechanism for the aerobic photo-oxidative dehydrogenation of urea-protected benzylamines to ketones **11**.

The proposed catalytic pathway begins with the generation of the excited photocatalyst 10* under blue light irradiation, producing the cation radical of the starting urea and the anion radical **10**^−•^ by SET (Scheme 6) [77]. Next, another SET process allows the recovery of photocatalyst **10** and the formation of the reduced superoxide radical ion O_2_^−•^ from the O_2_. This superoxide radical abstracts a proton and coordinates with the urea moiety, directing the addition to the imine formed in the process. Finally, the elimination of H_2_O leads to the formation of ketone **11** and a *N*-aryl urea.

**Scheme 7:**
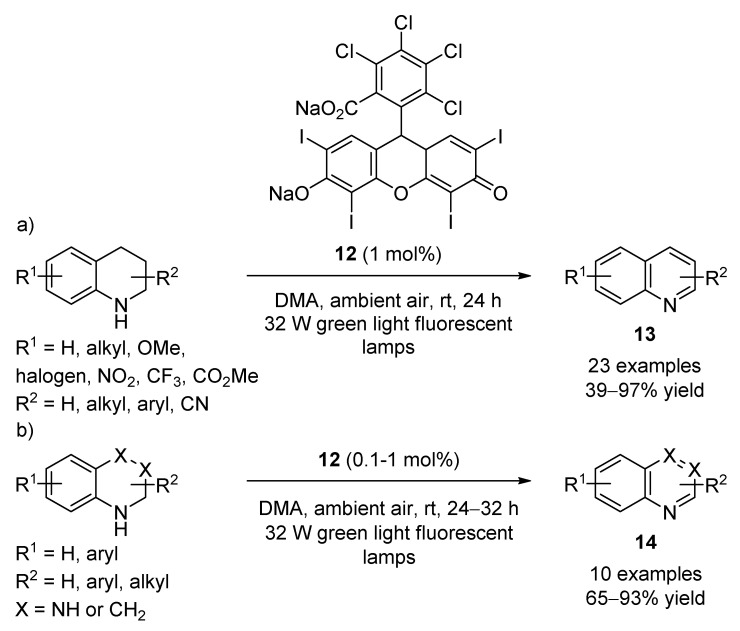
Aerobic photo-oxidative dehydrogenation of (**a**) tetrahydroisoquinolines and (**b**) other *N*-heterocycles to render **13**/**14** catalyzed by **12**.

The photocatalytic oxidative dehydrogenation of *N*-heterocycles is another interesting reaction that has been explored due to the biological interest in the obtained products [78]. Hence, an attractive synthetic methodology under green light irradiation using the xanthene-based organic dye Rose Bengal disodium salt (**12**) as photocatalyst was developed for the oxidative dehydrogenation of tetrahydroquinolines under base- and additive-free conditions in *N*,*N*-dimethylacetamide (DMA) as solvent, rendering the corresponding quinolines **13** in moderate to excellent yields (Scheme 7a) [79]. In order to expand the scope of the reaction using this direct synthetic route, other *N*-heteroaromatics **14,** such as acridines, benzofused-quinolines, quinoxalines and quinazolines, were synthesized in high yields from the corresponding starting compounds (Scheme 7b) [79].

**Scheme 8:**
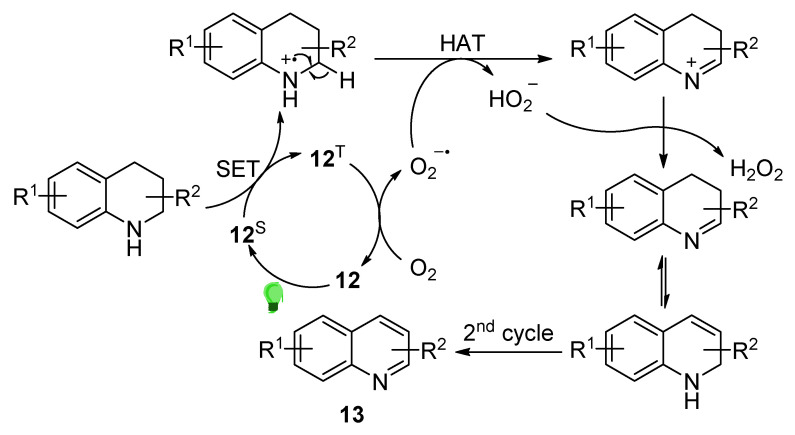
Proposed mechanism for the photo-organocatalytic oxidative dehydrogenation of tetrahydroquinolines to quinolines **13**.

The proposed mechanism is the same for **13** and **14** (Scheme 8) [79]. Thus, Rose Bengal disodium salt (**12**) is excited by green light irradiation to render it its singlet state **12**^S^, which oxidizes the amine by a SET process generating an amine radical cation while the triplet state of Rose Bengal **12**^T^ is formed (Scheme 8). At the same time, O_2_ is reduced to the superoxide radical ion that transforms the amine radical cation to an imine by a hydrogen atom transfer process (HAT), recovering the photocatalyst and generating hydrogen peroxide. The final step consists of the isomerization of the imine, after which a second catalytic cycle is produced to yield the desired product **13**.

**Scheme 9:**
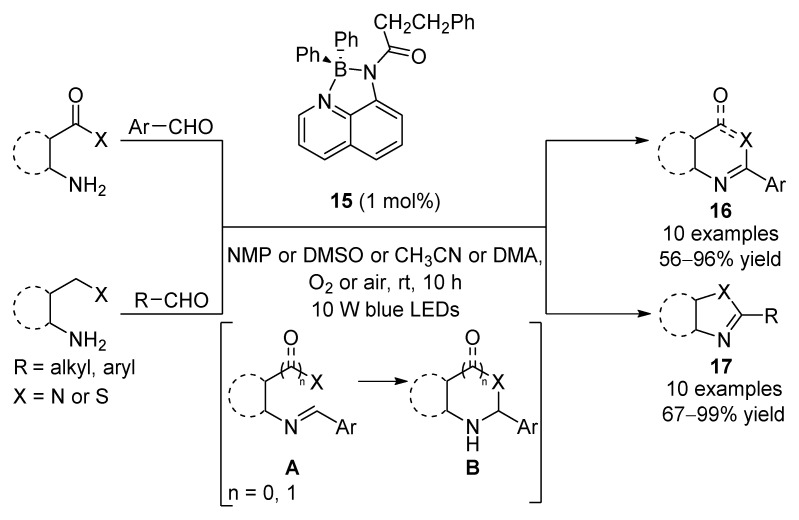
Aerobic photo-oxidative dehydrogenation of in situ generated *N*-heterocycles **B** catalyzed by **15**.

Recently, an aminoquinolate diarylboron compound **15**, which demonstrated good activity in the dehydrogenation of tetrahydroquinolines and other *N*-heterocycles leading to **13**/**14** [80], was used as a photocatalyst in the dehydrogenation of the products **B** generated from the cyclization of 2-substituted anilines with benzaldehydes to the intermediates **A**. Final **16**/**17** were obtained in moderate to excellent yields in *N*-methyl-2-pyrrolidone (NMP), dimethylsulfoxide (DMSO), CH_3_CN or DMA as solvent (Scheme 9) [80].

**Scheme 10:**
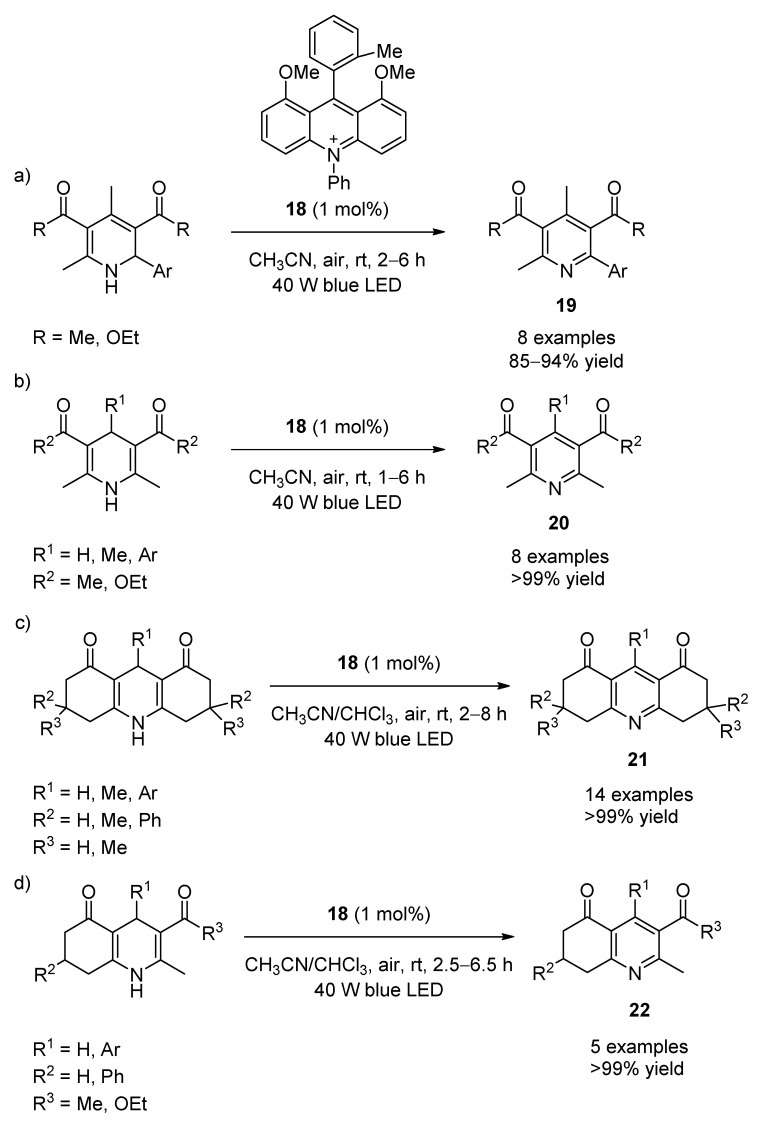
Aerobic photo-oxidative aromatization of: (**a**,**b**) dihydropyridines, (**c**) hexahydroacridinediones and (**d**) polyhydroquinolines, catalyzed by **18**.

Other *N*-heterocyclic compounds oxidized under aerobic conditions and irradiation with visible light were 1,2- and 1,4-dihydropyridines (Scheme 10a,b), hexahydroacridinediones (Scheme 10c) and polyhydroquinolines (Scheme 10d), employing a 1,8-dimethoxy-substituted acridinium ion **18** as catalyst, leading to the corresponding aromatic *N*-heterocycles **19**–**22** in excellent yields [81].

**Scheme 11:**
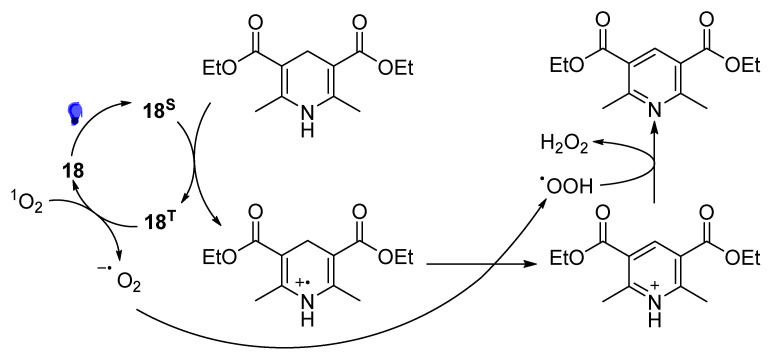
Proposed mechanism for the aerobic photo-oxidative aromatization of 1,4-dihydropyridines.

The proposed mechanism for these transformations, exemplified in the case of dihydropyridine, is shown in Scheme 11. First, photocatalyst **18** is excited to its singlet state **18^S^** by irradiation with visible light. Subsequently, a cation radical is generated in the nitrogen of the heterocyclic compound by a SET process, while the triplet state of the catalyst **18^T^** is formed. Finally, the cation radical reacts with the superoxide anion radical from the O_2_, recovering the starting photocatalyst and generating the corresponding cation, which is oxidized into the corresponding aromatic compound.

From all these results, it can be observed that, as supposed, lower photocatalyst loadings generally result in slower reactions. However, photocatalysts **8**, **15** and **18** yielded very good results in much shorter reaction times, despite low catalyst loading. These differences can be attributed to a higher photoabsorptivity of the photocatalysts and their ability to generate ROS, final yields materializing particularly well when using **8** and **18**.

### 2.2. Heterogeneous Oxidative Dehydrogenation of C–N Bonds

**Scheme 12:**
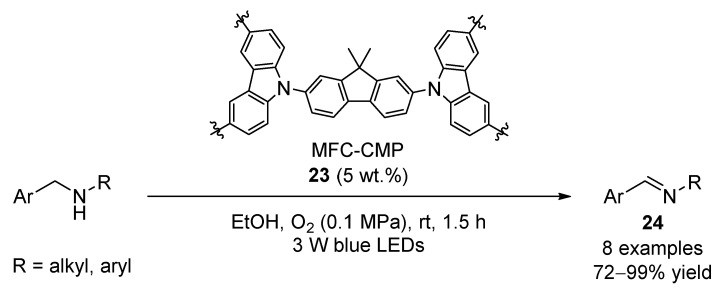
Aerobic photo-oxidative dehydrogenation of secondary amines to imines **24** catalyzed by **23**.

The aerobic dehydrogenation of amines to imines has also been carried out using metal-free heterogeneous photocatalysts under environmentally friendly conditions. These methodologies are very attractive, the catalysts being suitable for recycling using several procedures. For example, conjugated microporous polymers (CMPs) can present photoactive structures and high thermal stability and can be applied to several photocatalytic processes [82,83,84]. Thus, a carbazole-based CMP with a fluorene moiety (MFC-CMP, **23**) was recently employed in the aerobic dehydrogenation of secondary amines, leading to imines **24** in good to excellent yields (Scheme 12) [85]. The catalyst was reused in four cycles without losing its activity, although the recycling procedure was not described.

The proposed mechanism of transformation is the same as shown in previous homogeneous C–N bond oxidation (Scheme 1). The authors suggest that the formation of electrons at the electron donor moiety of carbazole and holes at the electron acceptor moiety of fluorine generates the superoxide anion radical and the amine cation radical.

**Figure 2:**
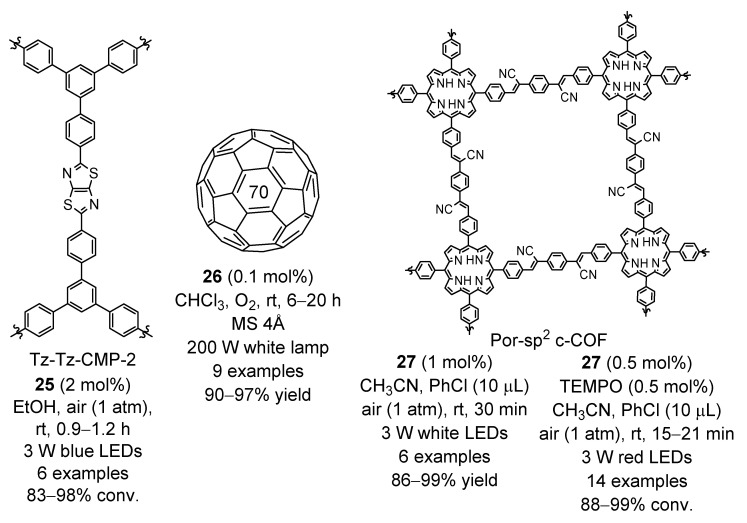
Results obtained in the aerobic oxidative dehydrogenation of secondary amines to imines **24** catalyzed by **25**–**27**.

Another CMP with a thiazolo [5,4-*d*]thiazole base structure (Tz-Tz-CMP-2, **25**) has been used in the same transformation, achieving excellent conversions to imines **24** (Figure 2) [86]. In addition, other heterogeneous catalysts, such as C_70_ fullerene (**26**) [87] and a 2D porphyrin-based sp^2^ carbon-conjugated covalent organic framework (Por-sp^2^ c-COF, **27**) [88] have demonstrated excellent activity towards the photocatalytic aerobic dehydrogenation of secondary amines to imines **24** (Figure 2). In the case of **27**, the authors published a later work in which the same heterogeneous photocatalyst was applied in a cooperative photocatalysis with TEMPO, also obtaining excellent results (Figure 2) [89]. Regarding the recyclability of these catalysts, the recycling of **25** was not mentioned by the authors, whereas **26** could be recovered and reused by precipitation from MeOH, obtaining a C_70_ fraction that contains C_70_ epoxides, which are then thermally treated to recover the underivatized C_70_. In the case of **27**, it could be recovered by centrifugation. 

**Figure 3:**
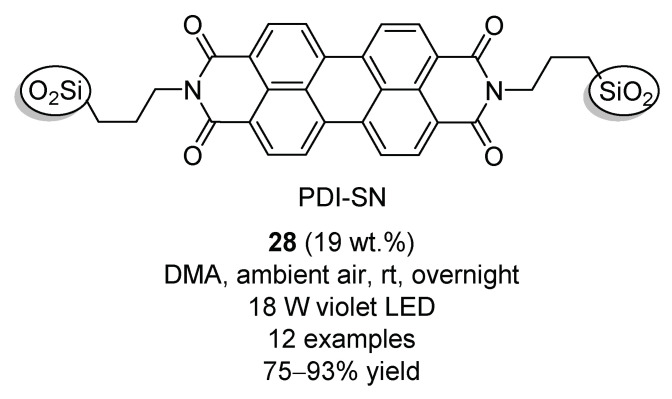
Results obtained in the photo-oxidative dehydrogenation of tetrahydroquinolines and other *N*-heterocycles to render **13** and **14** catalyzed by **28**.

The mentioned metal-free photocatalyzed oxidative dehydrogenation of *N*-heterocycles is another C–N bond oxidation reaction that has been performed under heterogeneous conditions. Thus, an environmentally friendly protocol, using a perylene diimide organic semiconductor covalently immobilized to SiO_2_ nanospheres (PDI-SN, **28**) as catalyst and DMA as solvent, was developed to carry out the oxidative dehydrogenation of tetrahydroquinolines and other *N*-heterocycles to render **13**/**14**, under violet light irradiation, in good to excellent yields (Figure 3) [90]. The photocatalyst **28** could be recycled up to six cycles, by washing the crude reaction mixture with acetone three times and drying the filtrate at 60 °C for 6 h, and reused without observing a significant decrease in its activity.

Recently, an imidazole-linked porphyrin-based CMP was employed in the aerobic oxidative dehydrogenation of *N*-heterocycles to render **13**/**14** in high yields [91]. The recyclability of the photocatalyst was carried out, and it was reused up to six cycles without remarkable changes in its activity.

**Scheme 13:**
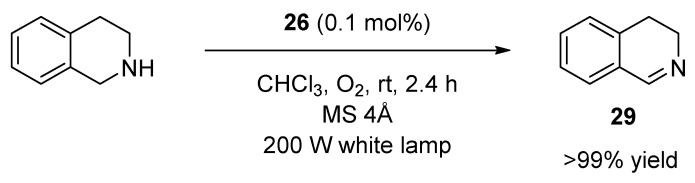
Partial aerobic photo-oxidation of 1,2,3,4-tetrahydroisoquinoline catalyzed by **26**.

Additionally, C_70_ fullerene (**26**) was also used for the partial aerobic oxidation of 1,2,3,4-tetrahydroisoquinoline under irradiation with white light and chloroform as solvent, which was previously saturated with oxygen, rendering the corresponding oxidized product **29** in quantitative yield (NMR) (Scheme 13) [87]. Furthermore, the same imidazole-linked CMP used in the dehydrogenation of *N*-heterocycles to **13**/**14** was used in the partial oxidation of 1,2,3,4-tetrahydroisoquinolines, leading to the corresponding isoquinolines in high yields [91]. The photocatalyst **26** was recycled by precipitation, whereas the CMP was recovered by centrifugation and reused in up to 10 cycles, both maintaining the activity.

**Scheme 14:**
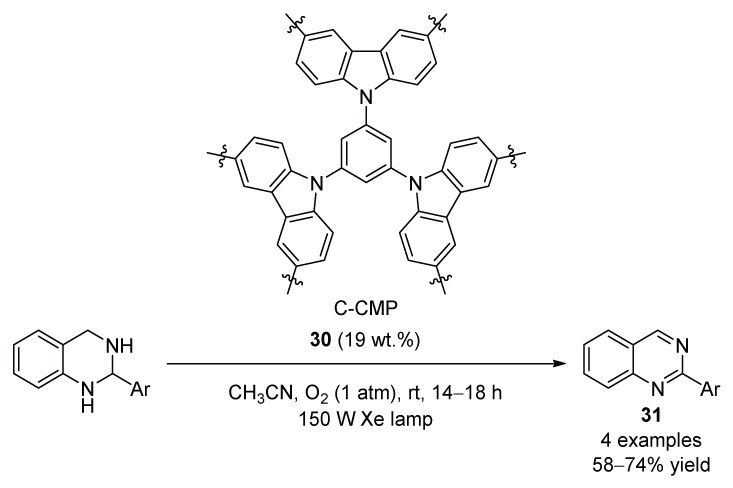
Aerobic photo-oxidation of tetrahydroquinazolines to quinazolines **31** catalyzed by **30**.

Carbazolic CMP (C-CMP, **30**, 15 wt.%), which was also used in the photo-oxidation of a secondary amine shown in Scheme 12 (Ar = Ph, R = Bn, 10 wt.%, 76% yield), was employed in the former partial aerobic photo-oxidation of tetrahydroisoquinoline, working in acetonitrile under irradiation with a 150 W Xe lamp at 10 °C, obtaining **29** in 72% yield [92]. In the same work, this photocatalyst was also employed in the aerobic oxidation of tetrahydroquinazolines to quinazolines **31** in moderate to good yields (Scheme 14). The catalyst **30** was reused, but not for this particular transformation.

**Figure 4:**
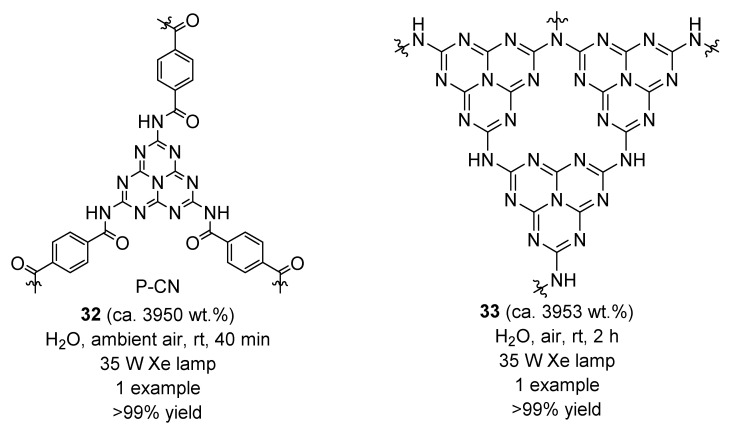
Results obtained in the photo-oxidative aromatization of the Hantzsch ester, leading to **20** catalyzed by **32** and **33**.

Two efficient and mild protocols using a polymeric carbon nitride with terephthalic acid monomers in its structure (P-CN, **32**) (Figure 4) [93] and a melamine-based carbon nitride **33** (Figure 4) [94] as photocatalysts, were developed for the aerobic photo-oxidative aromatization of a 1,4-dihydropyridine (Hantzsch ester), leading to the corresponding aromatic pyridine **20** (R^1^ = H, R^2^ = OEt) in quantitative yield. The catalyst **32** could be recycled three times without any change in its activity, while the reusability of **33** was not reported.

**Figure 5:**
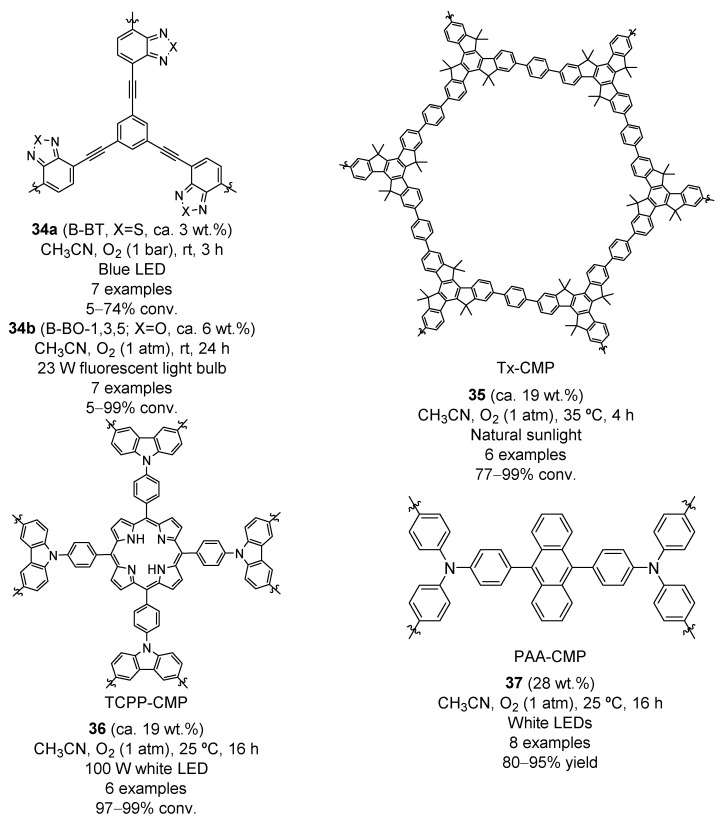
Results obtained in the aerobic photo-oxidative coupling of benzylamines to *N*-benzylidene benzylamines **2** catalyzed by **34**–**37**.

The aerobic oxidative coupling of benzylamines under irradiation with visible light (Scheme 2) has been widely explored using polymeric photocatalysts. CMPs are one of those polymer-based catalysts studied for this transformation. Thus, a benzothiadiazole-based CMP (B-BT, **34a**) and a benzooxadiazole-based CMP (B-BO-1,3,5; **34b**) were used in the mentioned reaction, obtaining good results (Figure 5) [95,96]. In both cases, the catalyst was recovered for up to five cycles with only a slight decrease on the activity of **34b**, the authors not reporting the recycling procedure. 

**Figure 6:**
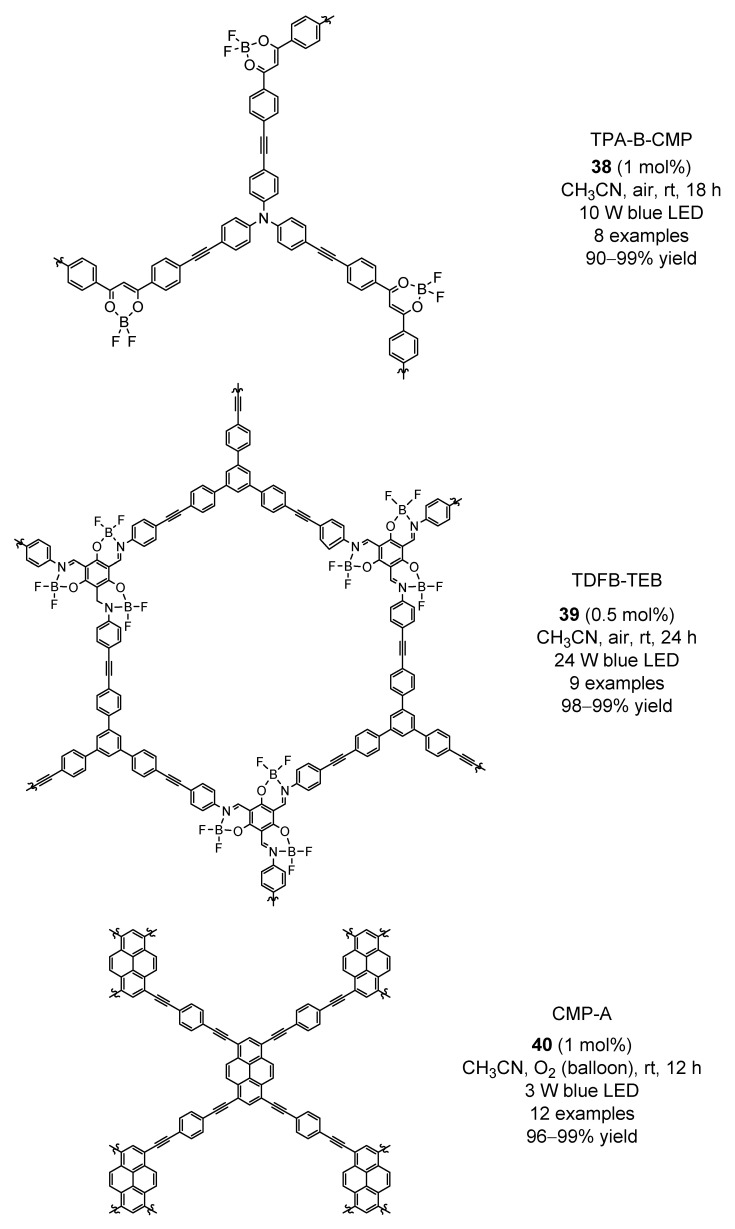
Results obtained in the aerobic photo-oxidative coupling of benzylamines to *N*-benzylidene benzylamines **2** catalyzed by **38**–**40**.

Other CMPs employed as photocatalysts in the same reaction leading to **2** are a truxene-based polymer (Tx-CMP, **35**) under natural sunlight irradiation [97], a carbazolyl porphyrin-based CMP (TCPP-CMP, **36**), also employed in the aerobic dehydrogenation of amines to imines shown in Scheme 12 (Ar = Ph, R = ^i^Pr, Bn, CH_2_-(4-MeOC_6_H_4_), ca. 19 wt.%, CH_3_CN, O_2_ (1 atm), 25 °C, 16 h, 100 W white LED, 3 examples, 99% conv.) [98], a triphenylamine-based CMPs functionalized with anthracene moieties (PAA-CMP, **37**) [99] (Figure 5) and with β-diketone boron difluoride motifs (TPA-B-CMP, **38**) [100], a boranil-based polymeric material (TDFB-TEB, **39**) [101] and a pyrene-based polymeric promoter (CMP-A, **40**) [102] (Figure 6). In all cases, excellent results were obtained. It should be noted that all the catalysts could be recovered by centrifugation, washing and drying (**35**, **38** and **39**), by simple centrifugation (**36** and **37**) or by filtration (**40**), maintaining their activity for five/six cycles without any noticeable change in performance.

**Figure 7:**
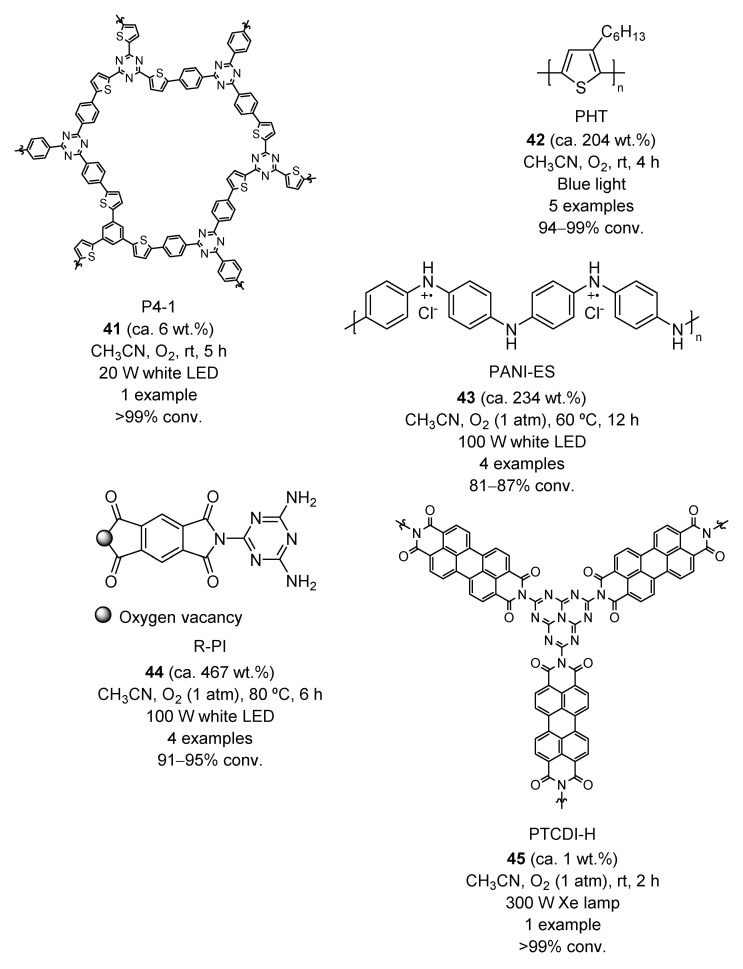
Results obtained in the aerobic photo-oxidative coupling of benzylamines to *N*-benzylidene benzylamines **2** catalyzed by **41**–**45**.

Other conjugated polymers have also been used in this transformation leading to **2**. Thus, a conjugated trithienyl triazine-based polymer (P4-1, **41**) [103], conjugated polyhexylthiophene nanofibers (PHT, **42**) [104], an emeraldine salt of a conjugated HCl-doped polyaniline (PANI-ES, **43**) [105] and two conjugated imide-based polymers, an oxygen vacancies-rich polyimide (R-PI, **44**) [106] and ultrathin nanosheets containing imide motifs (PTCDI-H, **45**) [107], have been successfully employed as heterogeneous photocatalysts (Figure 7). In the case of the reactions promoted by **43** and **44**, butylamine was also tried, albeit without success. All photocatalysts could be recycled five times (for **41**, **44** and **45**) and six times (for **42** and **43**) by centrifugation without observing significant variations in their activity in most cases, although polyaniline **43** needed to be regenerated with HCl, with photocatalytic performance dropping between cycles due to leaching.

**Figure 8:**
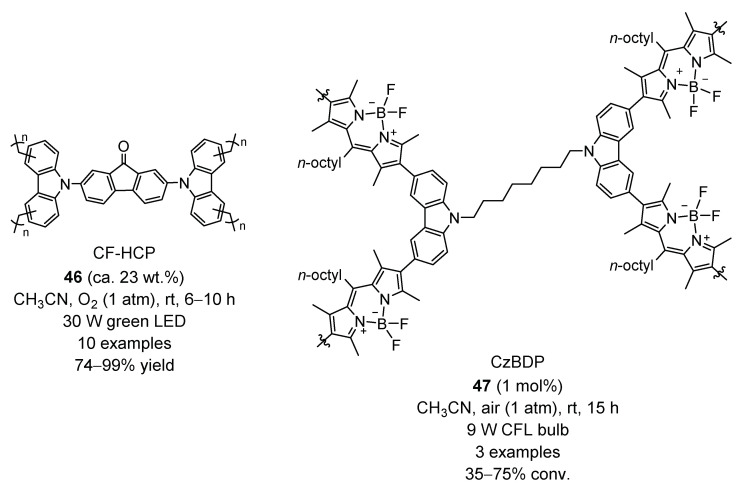
Results obtained in the aerobic photo-oxidative coupling of benzylamines to *N*-benzylidene benzylamines **2** catalyzed by **46** and **47**.

Porous organic polymers (POPs), which have different applications in photocatalysis [108], have also been used as heterogeneous photocatalysts in the same oxidative coupling of benzylamines. In this sense, two carbazole-based POPs were recently used in the aforementioned reaction, the fluorenone-based (CF-HCP, **46**) [109] and the BODIPY-based (CzBDP, **47**) [110], successfully obtaining the corresponding *N*-benzylidene benzylamines **2** (Figure 8). It is worth mentioning that **46** was also employed in the aerobic dehydrogenation of secondary amines to the imines **24** shown in Scheme 12 (Ar = Ph, R = Bn, ^t^Bu, ca. 23 wt.%, CH_3_CN, O_2_ (1 atm), 25 °C, 6–10 h, 30 W green LED, 2 examples, 81–93% yield), as well as in the partial aerobic oxidation of the 1,2,3,4-tetrahydroisoquinoline shown in Scheme 13 (ca. 23 wt.%, CH_3_CN, O_2_ (1 atm), 25 °C, 10 h, 30 W green LED, >99% yield) [109]. The recycling of **46** was not carried out for this particular transformation, while **47** was not reused.

**Figure 9:**
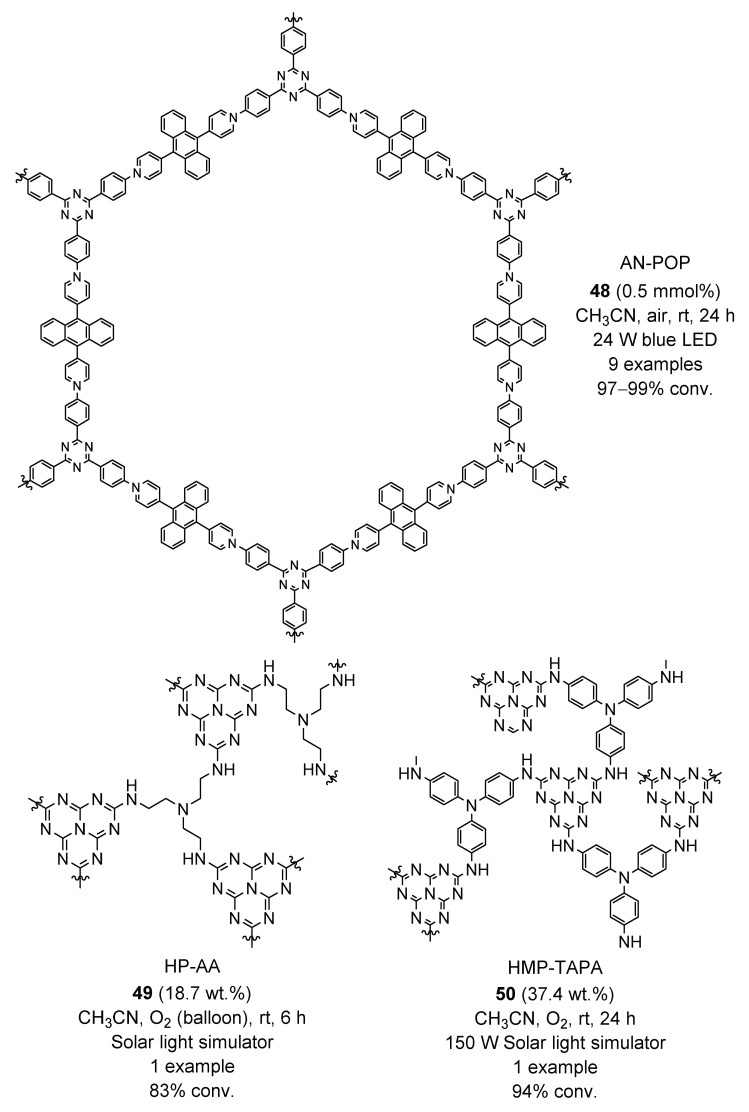
Results obtained in the aerobic photo-oxidative coupling of benzylamines to *N*-benzylidene benzylamines **2** catalyzed by **48**–**50**.

In addition, a hyper-cross-linked polymeric material, synthesized via the AlCl_3_-catalyzed coupling reaction of hexaphenylbenzene monomers, was used with irradiation with a 210 W Xe lamp under air, obtaining moderate conversions to **2** [111], while the use of an anthracene ionic polymer (AN-POP, **48**) [112] and two heptazine-based polymeric promoters, presenting tris-aminoethylamine units (HP-AA, **49**) [113] and tris(4-aminophenyl)amine moieties (HMP-TAPA, **50**) [114], allowed for the obtention of **2** in quantitative conversions (Figure 9). In the case of the hyper-cross-linked polymer, it was also tested in the aerobic dehydrogenation of secondary amines to the imines **24** shown in Scheme 12 (Ar = Ph, R = Bn, ca. 19 wt.%, CH_3_CN, O_2_ (1 atm), 25 °C, 4 h, 210 W Xe lamp, 75% yield) [111]. The reusability of the photocatalysts was studied by filtration and washing with CH_3_CN (for **48**), and centrifugation (for **49** and **50**), without observing remarkable changes in their activity.

**Figure 10:**
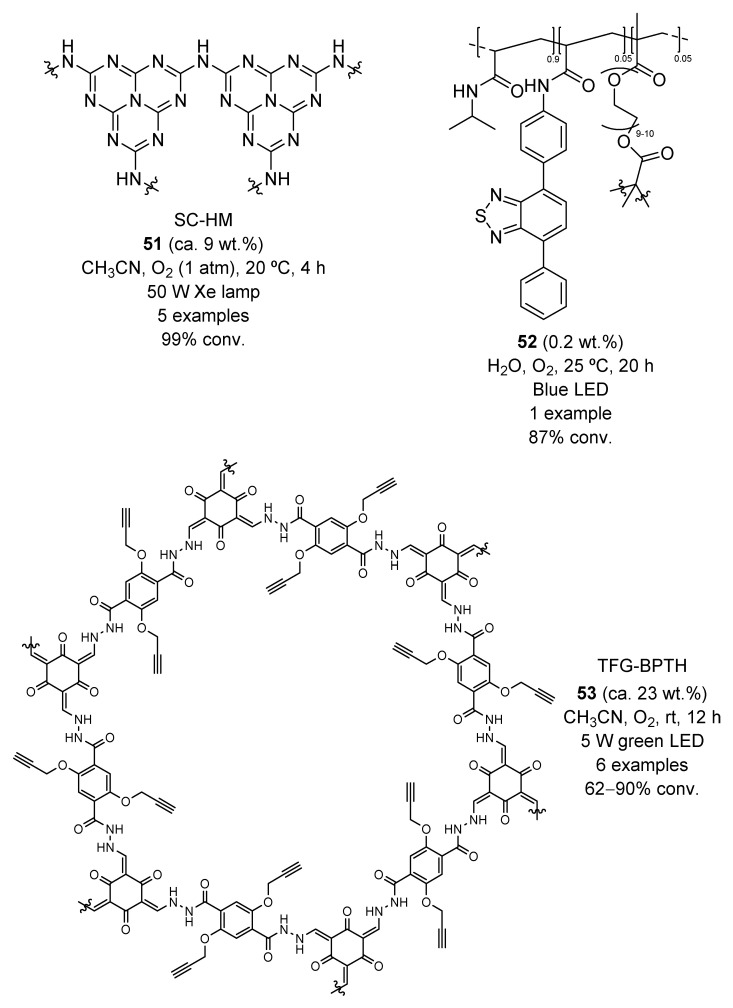
Results obtained in the aerobic photo-oxidative coupling of benzylamines to *N*-benzylidene benzylamines **2** catalyzed by **51**–**53**.

Other polymers recently employed in the same reaction leading to **2** were a melamine-based material (SC-HM, **51**) [115], a nanogel composed of poly(*N*-isopropylacrylamide), *N*-(4-(7-(phenylbenzo[*c*][1,2,5]thiadiazol-4-yl)phenyl)acrylamide and polyethyleneglycol dimethylacrylate monomers **52** [116] and an organic polymer based on 1,3,5-triformylphloroglucinol and a terephthalohydrazide (TFG-BPTH, **53**) [117]. These promoters enabled the preparation of *N*-benzylidene benzylamines **2** (for **52**: Ar = Ph) in moderate to good conversions in all cases (Figure 10). The recyclability was tested in another photo-oxidation reaction (for **51** and **52**) and by filtration (for **53**), without any loss in their catalytic activity.

**Figure 11:**
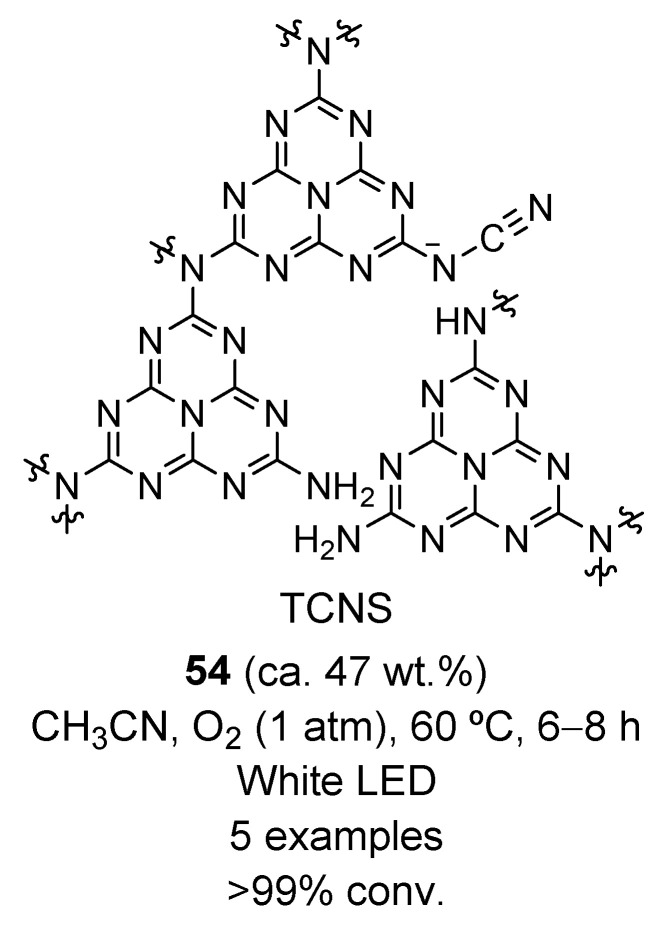
Results obtained in the aerobic photo-oxidative coupling of benzylamines to *N*-benzylidene benzylamines **2** catalyzed by **54**.

Polymeric carbon nitride as a metal-free promoter has been used in many photoredox processes [118]. Thus, the former carbon nitride **33** obtained from cytosine and urea units [119] and a cyanated modified carbon nitride (TCNS, **54**) (Figure 11) [120] were employed in the aerobic oxidative homocoupling of benzylamines leading to **2**, achieving moderate to excellent conversions. This latter catalyst was recovered and reused up to four times without loss of performance, but the recycling procedure was not specified. In another work leading to **2**, acetone was needed as co-catalyst to promote the spin relaxation and cancel non-radiative energy losses, using **33** as photocatalyst (ca. 4 wt.%, 10 µL acetone, CH_3_CN, O_2_ (1 atm), 20 °C, 3 h, Xe lamp, 8 examples, 90–99% conv.) [121]. Additionally, a polymeric carbon nitride (93.5 wt.%), organized in layers and synthesized by a self-assembly approach, introducing hydroxyl and carboxyl groups, was employed in the aerobic oxidative coupling of benzylamine leading to *N*-benzylidene benzylamine in 58% yield [122].

The mentioned carbon nitride **33** packed in a flow reactor, which was also used in the photo-oxidative aerobic dehydrogenation of secondary amines shown in Scheme 12 (Ar = Ph, R = Bn) with a conversion to **24** of 78%, allowed for the obtention of **2** in excellent conversions (ca. 4 wt.%, CH_3_CN, air, 55 °C, τ = 60 min, 14.4 W blue LED, 5 examples, 95–100% conv.) [123].

**Figure 12:**
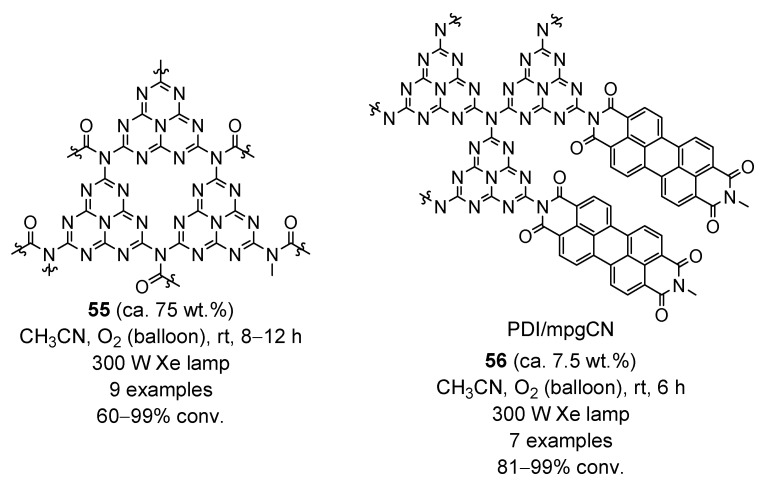
Results obtained in the aerobic photo-oxidative coupling of benzylamines to *N*-benzylidene benzylamines **2** catalyzed by **55** and **56**.

Graphitic carbon nitrides, which have been extensively applied in many photocatalytic processes related to energy and environment [124,125,126], have been recently used in the mentioned oxidative coupling leading to **2**. Recently reported examples of the use of this type of metal-free photocatalysts include a ketone-group modified graphitic carbon nitride **55**, which successfully catalyzed this transformation (Figure 12) [127], also showing excellent activity in the partial photo-oxidation of 1,2,3,4-tetrahydroisoquinoline in Scheme 13, as well as **54** in its graphitic form (ca. 234 wt.%, CH_3_CN, O_2_ (1 atm), 25 °C, 4 h, 300 W Xe lamp, 9 examples, 94–99% conv.) [128]. In addition, a garland-like interconnected graphitic carbon nitride, synthesized via an oxalic acid-mediated assembly strategy, was also employed, obtaining good results (ca. 47 wt.%, CH_3_CN, O_2_ (1 atm), 20 °C, 8–33 h, 300 W Xe lamp, 9 examples, 82–99% conv.) [129], while a perylenetetracarboxylic diimide covalently connected in a graphitic carbon nitride (PDI/mpgCN, **56**) promoted the reaction in excellent conversions (Figure 12) [130]. Concerning recovery, the performance of **55** was maintained with a slight decrease in the third reaction cycle, which was overturned by washing the catalyst with a 0.1M solution of NaOH. The graphitic form of **54** was reused by centrifugation, while **56** was recycled by filtration without a noticeable drop in their activity. The recyclability of the garland-like graphitic carbon nitride was not tested for this transformation.

In addition, a porous few-layer carbon nitride obtained from melamine and cyanuric acid by molecular self-assembly was used to render *N*-benzylidene benzylamines **2** in moderate to excellent conversions (ca. 4 wt.%, CH_3_CN, O_2_ (0.1 MPa), 25 °C, 1 h, 300 W Xe lamp, 7 examples, 82–99% conv.) [131]. The catalyst could be recovered and reused for up to five cycles without decreasing its activity. 

**Figure 13:**
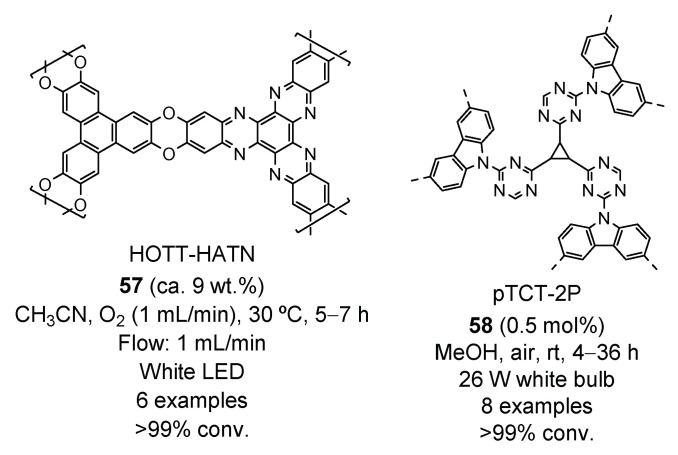
Results obtained in the aerobic photo-oxidative coupling of benzylamines to *N*-benzylidene benzylamines **2** catalyzed by **57** and **58**.

**Figure 14:**
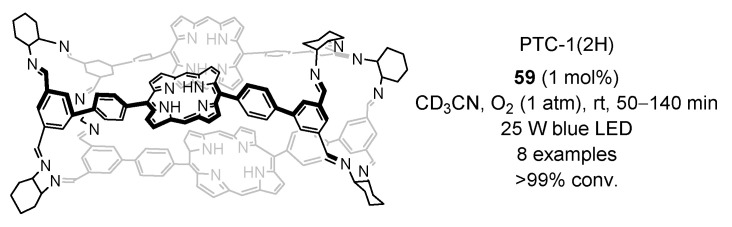
Results obtained in the aerobic photo-oxidative coupling of benzylamines to *N*-benzylidene benzylamines **2** catalyzed by **59**.

Porous organic frameworks (POFs) have also been employed in the aerobic oxidative coupling of benzylamines leading to **2**, a few examples being recently published. In this sense, a nanoporous graphene-like POF synthesized from hexahydroxytriphenylene and hexaazatrinaphthylene monomers (HOTT-HATN, **57**) [132], a carbazole-triazine based donor–acceptor POF (pTCT-2P, **58**) [133] (Figure 13) and a supramolecular microporous porphyrin-based POF (PTC-1(2H), **59**) [134] (Figure 14) were used as photocatalysts in the mentioned transformation, obtaining excellent conversions and working under flow conditions in the first case. All these catalysts were recovered by filtration and reused for five cycles, maintaining their catalytic activity.

**Figure 15:**
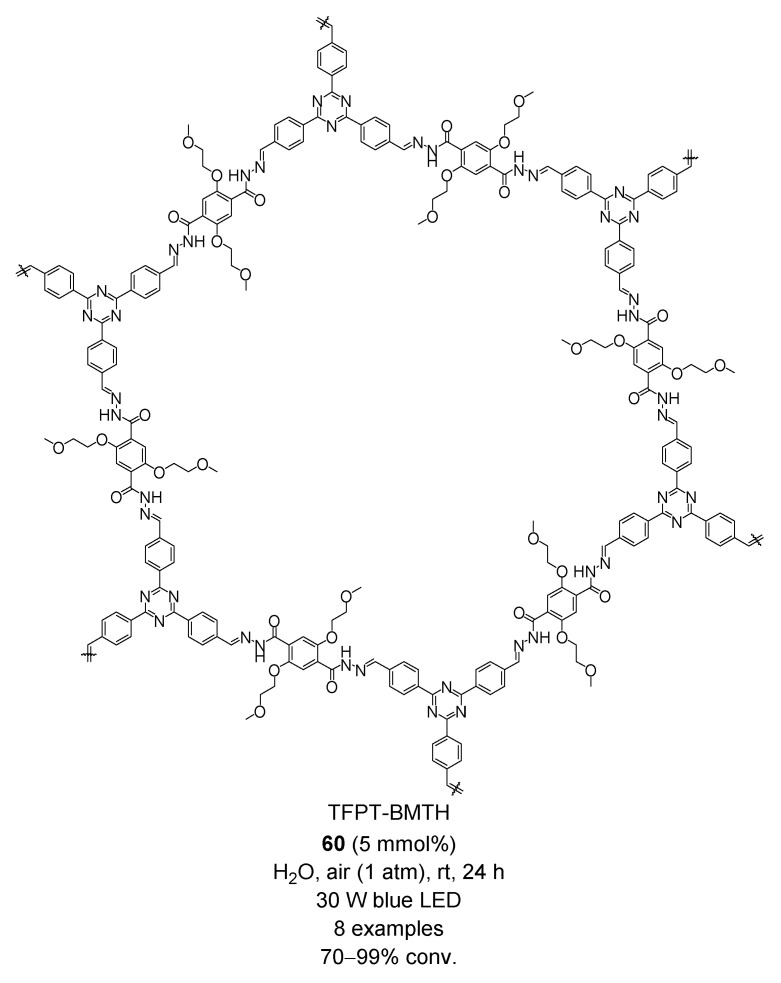
Results obtained in the aerobic photo-oxidative coupling of benzylamines to *N*-benzylidene benzylamines **2** catalyzed by **60**.

Covalent organic frameworks (COFs) have also been used in the aforementioned reaction. Thus, a hydrophilic hydrazone-based COF with 2-methoxyethoxy groups (TFPT-BMTH, **60**) has been employed as photocatalyst, yielding **2** in moderate to excellent conversions (Figure 15) [135]. The promoter was recovered by filtration without any change in its activity.

**Figure 16:**
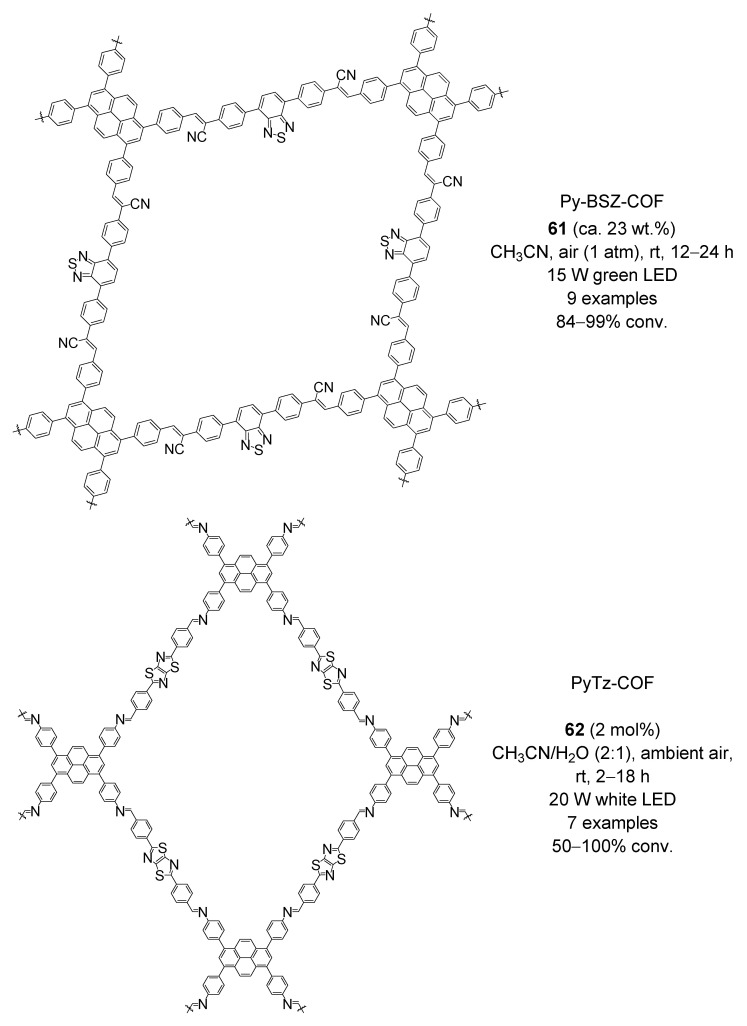
Results obtained in the aerobic photo-oxidative coupling of benzylamines to *N*-benzylidene benzylamines **2** catalyzed by **61** and **62**.

Other COFs used successfully in the same transformation were a pyrene-benzothiadiazole conjugated donor–acceptor (Py-BSZ-COF, **61**) [136] and a pyrene-thiazolo[5,4-*d*]thiazole donor–acceptor (PyTz-COF, **62**) [137] (Figure 16). The recyclability of these photocatalysts was achieved by centrifugation in both cases, being able to perform the reaction four and six times, respectively, maintaining their performance.

**Figure 17:**
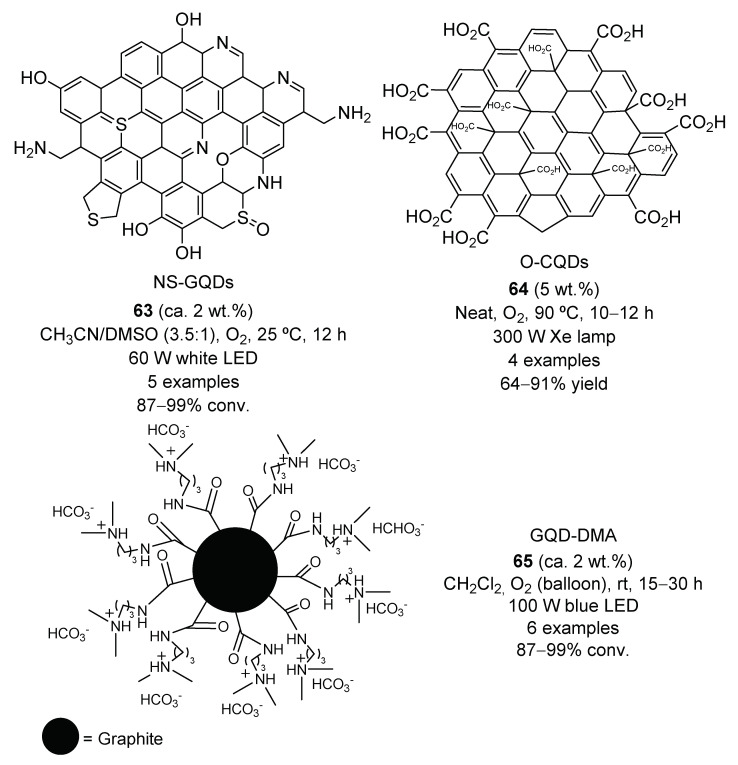
Results obtained in the aerobic photo-oxidative coupling of benzylamines to *N*-benzylidene benzylamines **2** catalyzed by **63**–**65**.

Graphene-based composites have been recently employed in many photocatalytic processes, such as those related to the environmental area [138,139,140]. Metal-free graphene quantum dots (GQDs) and carbon quantum dots (CQDs) are interesting materials due to their unique physical and chemical properties, demonstrated to have various photocatalytic applications [141,142]. Thus, the aerobic oxidative coupling of benzylamines for the preparation of **2** has been successfully carried out with nitrogen- and sulfur-codoped GQDs synthesized from a catecholamine derivative (NS-GQDs, **63**) [143], oxygen-rich CQDs (O-CQDs, **64**) [144] and dimethylamino-functionalized GQDs (GQD-DMA, **65**) [145] (Figure 17). Recycling experiments were performed by precipitation (for **63**), vacuum distillation of the reactants, products and solvents (for **64**), and by CO_2_ bubbling for 10 min (for **65**), without observing significant changes in the results.

**Scheme 15:**
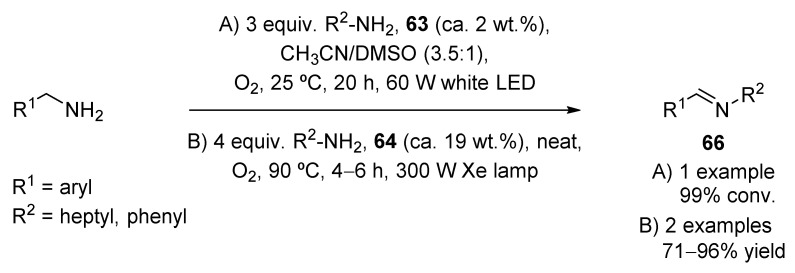
Aerobic photo-oxidative heterocoupling of amines catalyzed by **63** or **64**.

Remarkably, catalysts **63** and **64** were also able to effectively promote the amine aerobic photo-oxidative heterocoupling, yielding imines **66** in good yields (Scheme 15) [143,144].

**Figure 18:**
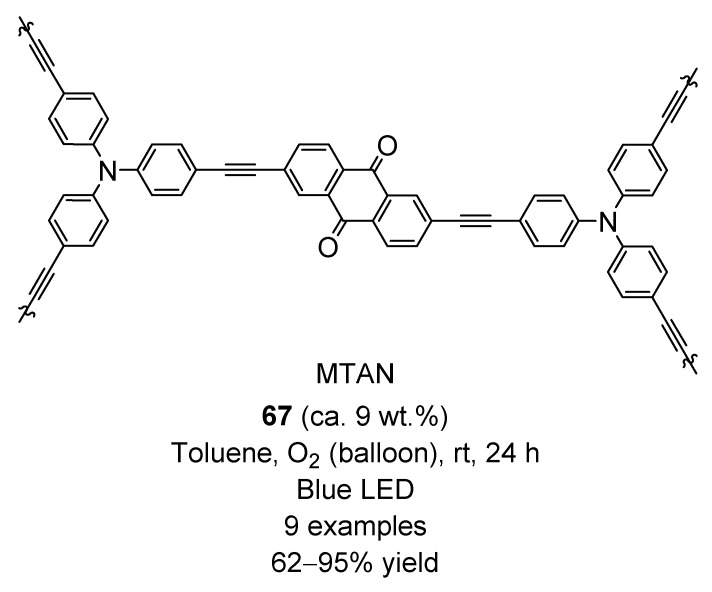
Results obtained in the aerobic photo-oxidative coupling of benzylamines to *N*-benzylidene benzylamines **2** catalyzed by **67**.

Another different type of photocatalyst employed in the aerobic photo-oxidative coupling of benzylamines was a hollow microporous organic network obtained from tris(4-ethynylphenyl)amine and 2,6-diiodo-9,10-anthraquinone (MTAN, **67**), rendering **2** in moderate to excellent yields (Figure 18) [146]. Besides this reaction, **67** was also employed in the aerobic photo-oxidative dehydrogenation of secondary amines shown in Scheme 12 (Ar = Ph, R = Bn, ca. 9 wt.%, toluene, O_2_ (balloon), rt, 24 h, blue LED, 55% yield) [146]. The photocatalyst was not reused.

In addition, the C_70_ fullerene **26** was also employed in the photo-oxidative homocoupling of benzylamines leading to **2** in excellent yields (0.05 mol%, CH_3_CN, O_2_, rt, 24 h, 34 W blue LED, 17 examples, 67–98% yield) [147], and also in the aerobic oxidative dehydrogenation of amines to yield aldehydes **9** (0.05 mol%, CHCl_3_, O_2_, rt, 24 h, 34 W blue LED, 14 examples, 37–96% yield) [147]. The recyclability of the photocatalyst was not tested.

From the above-presented results, it can be observed that the structural variety of the recently employed heterogeneous photocatalysts scores higher than in the case of their homogenous counterparts. When polymeric-based heterogeneous photocatalysts are employed, the reaction times are generally shorter, while the reaction yields or conversions are usually high, showing their good photoabsorptivity and capacity to generate ROS. In contrast, the reaction times are usually higher when POFs, COFs and graphene-based photocatalysts are used, while the yields and conversions are excellent. Nevertheless, the results can be considered excellent in almost all oxidative dehydrogenations shown. Considering the crucial point of recyclability, polymeric-based photocatalysts could generally be recycled more easily and in more catalytic cycles than other heterogeneous photocatalyst types, although good results were also obtained with other heterogeneous systems.

## 3. Oxidative Dehydrogenation of C–O Bonds

### 3.1. Homogeneous Oxidative Dehydrogenation of C–O Bonds

The selective oxidative dehydrogenation of alcohols to the corresponding carbonyl compounds (ketones, aldehydes, carboxylic acids, etc.) is one of the most important reactions in organic chemistry and has become a very active research area in recent years. To perform this transformation, the use of air or pure O_2_ as the terminal oxidant, non-toxic metal-free compounds as catalysts and mild conditions under visible light, is interesting from an environmental point of view, the development of effective, versatile and sustainable methodologies being desirable.

**Scheme 16:**
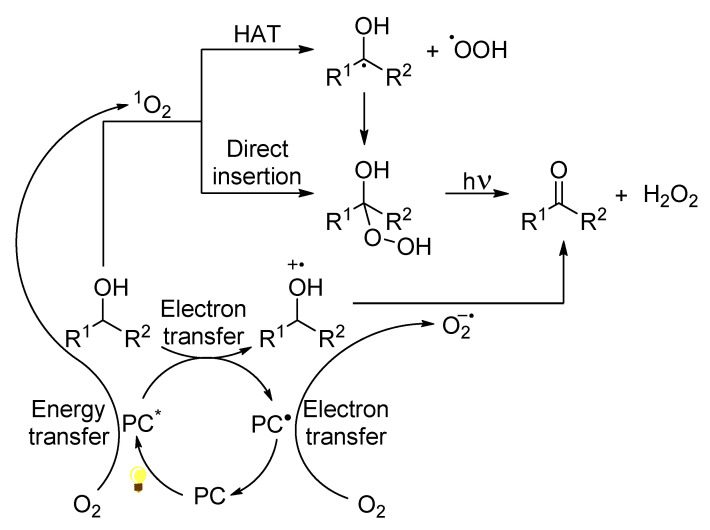
Commonly accepted mechanism of the aerobic photocatalytic oxidation of alcohols to carbonyl compounds.

The commonly accepted mechanism for the aerobic photocatalytic oxidative dehydrogenation of alcohols is shown in Scheme 16 [148]. Firstly, the photocatalyst (PC) is excited by irradiation with visible light to form its excited state (PC*), which is converted to its free radical state (PC^•^) by an electron transfer process, and the cation radical of the alcohol is formed. At the same time, an ET process is developed to produce the singlet oxygen (^1^O_2_) from the triple oxygen (^3^O_2_). The photocatalyst is recovered by another electron transfer process between the PC^•^ and the O_2_, obtaining the corresponding reduced superoxide radical ion (O_2_^−•^) that can oxidize the cation radical of the alcohol to the final carbonyl compound [149]. The singlet oxygen generated before could follow two pathways: the first one consists of a HAT process and radical recombination to form a peroxy hemiacetal intermediate that collapses to the final oxidized product, while in the second one, the singlet oxygen is directly inserted on the carbon bearing the hydroxyl group to form the mentioned peroxy hemiacetal that also collapses to the carbonyl compound [150]. In all the pathways, hydrogen peroxide is generated.

**Scheme 17:**
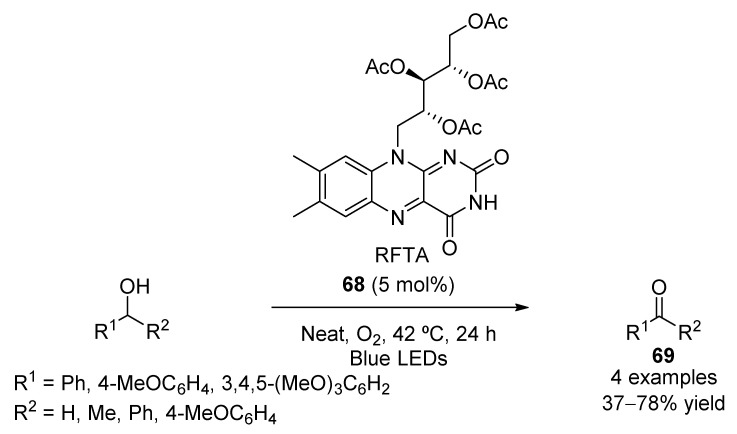
Aerobic photo-oxidation of primary and secondary alcohols, including fluorenol, catalyzed by **68**.

Flavin-based promoters are a promising type of photocatalyst that have been employed in different visible light-mediated transformations in recent years [151,152,153,154,155]. In this context, riboflavin tetraacetate (RFTA, **68**), derived from riboflavin (vitamin B2), could successfully catalyze the oxidation of different aromatic primary and secondary alcohols to the corresponding aldehydes and ketones **69** in the presence of molecular oxygen under visible light and solvent-free conditions (Scheme 17) [156].

**Figure 19:**
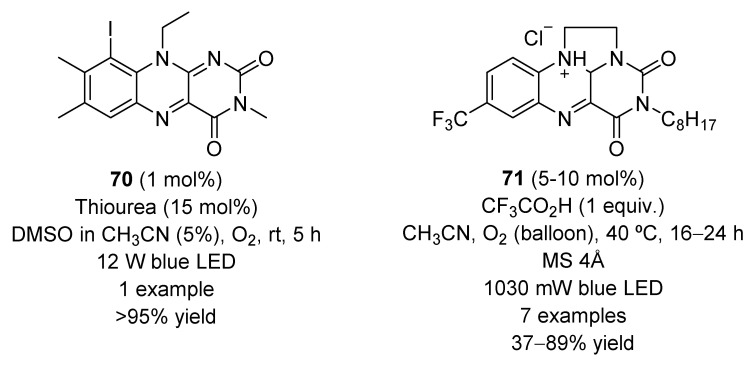
Results obtained in the aerobic photo-oxidation of aromatic alcohols catalyzed by **70** and **71**.

Other flavin-related compounds used as photocatalysts in the oxidation dehydrogenation of alcohols to **69** were **70**, which oxidized the benzyl alcohol in more than 95% yield using thiourea as electron mediator (Figure 19) [157], and the flavinium salt **71**, which could catalyze the oxidation of *para*-substituted aromatic alcohols to the corresponding carboxylic acids (**69**: R^1^ = Ph, 4-ClC_6_H_4_, 4-BrC_6_H_4_, 4-CF_3_C_6_H_4_; R^2^ = OH), aldehydes (**69**: R^1^ = 4-NO_2_C_6_H_4_; R^2^ = H) and ketones (**69**: R^1^ = 4-ClC_6_H_4_, 4-CF_3_C_6_H_4_; R^2^ = Me) in good yields using trifluoroacetic acid (CF_3_CO_2_H) as additive (Figure 19) [158].

**Figure 20:**
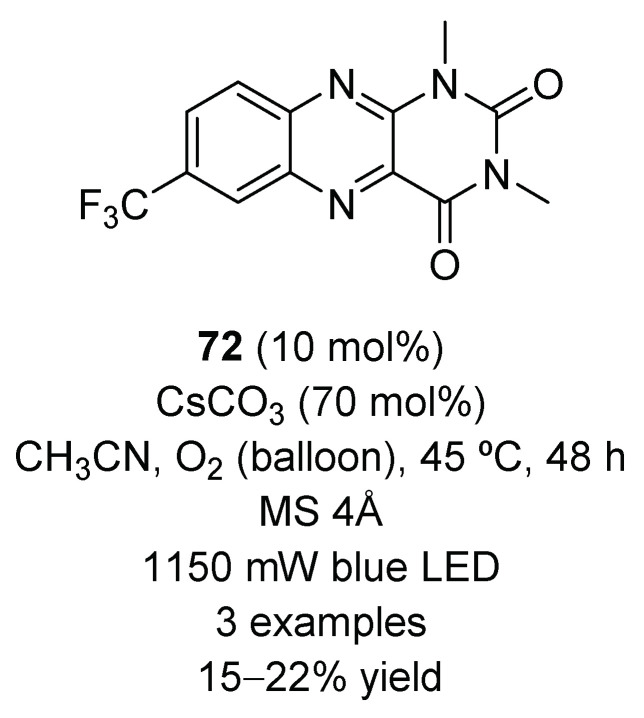
Results obtained in the aerobic photo-oxidation of aliphatic alcohols to ketones **69** catalyzed by **72**.

The flavinium salt **71** (5 mol%) was also recently employed in the oxidative dehydrogenation of aromatic secondary alcohols to render ketones using cesium carbonate (CsCO_3_, 70 mol%) as additive, O_2_ (balloon) as oxidant and under blue light irradiation, obtaining good yields [159]. In the same work, the authors reported another flavin-based photocatalyst **72** for the oxidation of cyclohexanol, 2-cyclohexenol and decan-2-ol to the corresponding ketones **69** under the same reaction conditions, although in low yields (Figure 20) [159].

Xanthene-based catalysts have been used in many photoredox reactions in recent years [160,161,162]. Recently, a neutral form of Rose Bengal **12** (5 mol%), in cooperation with NH_4_SCN (3 equiv.), could successfully catalyze the oxidative dehydrogenation of aromatic alcohols, including (9-ethyl-carbazol-3-yl)methanol, (indol-3-yl)methanol and cinnamyl alcohol to the corresponding aldehydes **69** in good to excellent yields [163].

Additionally, other xanthene-related photocatalysts used were Eosin Y (**3**, 1 mol%), which promoted the oxidation of aromatic and long-chain aliphatic primary alcohols to aldehydes **69** in moderate to good yields under O_2_ atmosphere (balloon) and irradiated with a 50 W Xe lamp [164]. Thioxanthone (5–20 mol%) also successfully catalyzed the oxidation of aromatic and aliphatic primary and secondary alcohols (including cinnamyl alcohol, 1,3-diphenylpropargylalcohol, xanthenol, ciclohexanol, borneol, menthol and cholesterol) to aldehydes or ketones **69**, obtaining excellent results [150]. The last approach was also applied, in the same work, to the synthesis of quinazolinones by oxidation of alcohols to aldehydes, imine formation using *ortho*-aminobenzamides, cyclization and final oxidative dehydrogenation of a C–N bond.

Other simple molecules have been employed as photocatalysts to carry out the aforementioned oxidation. Thus, metal-free TPP (0.005 mol%) promoted the oxidative dehydrogenation of aromatic and aliphatic alcohols to aldehydes and ketones **69** in low yields under solvent-free and ambient conditions [165]. In this reported strategy, epoxides of cinnamyl alcohol and cinnamaldehyde, and the hydroperoxide of the cycloheptanol, were generated as byproducts in the corresponding reactions. In addition, the use of 9-fluorenone as photocatalyst (3–6 mol%) allowed for the obtention of aldehydes or ketones **69** from alcohols (including xanthenol, cyclohexanol and heteroaromatic substrates) in moderate to excellent yields under blue light irradiation [149]. Moreover, tetrabutylammonium tribromide (TBATB, 13 mol%) promoted the formation of aldehydes and ketones **69** from alcohols in excellent isolated yields under violet or blue light irradiation [166], a low amount of the corresponding carboxylic acid being observed. Furthermore, sodium trifluoromethanesulfinate (5 mol%) catalyzed the oxidation of secondary alcohols to ketones **69** under violet light, obtaining very good results [167].

**Scheme 18:**
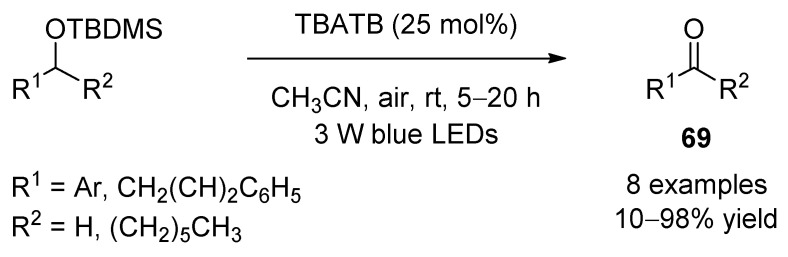
Aerobic photo-oxidation of primary and secondary *tert*-butyldimethylsilyl ethers catalyzed by TBATB.

TBATB has also been used as catalyst for the photo-oxidation of silyl ethers to aldehydes and ketones **69**, obtaining low to excellent yields (Scheme 18) [167].

**Scheme 19:**
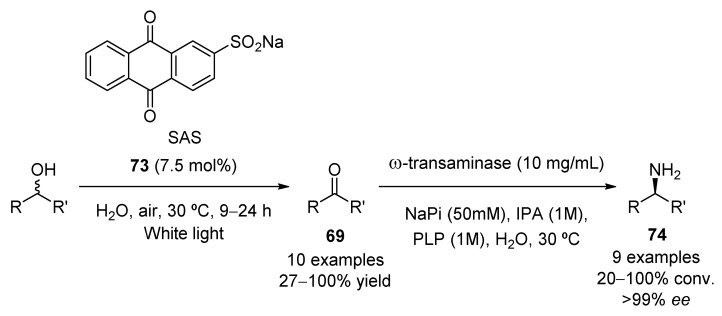
Cascade transformation of racemic alcohols into enantiopure amines **74** by oxidative dehydrogenation catalyzed by **73** and enantioselective transamination.

Anthracene-based promoters have also been employed to photocatalyze the oxidative dehydrogenation of alcohols to carbonyl compounds **69**. Thus, the water-soluble sodium anthraquinone-2-sulfonate (SAS, **73**, ca. 34 mol%) allowed for the obtention of aldehydes and ketones **69** from aromatic and aliphatic alcohols (including cyclohexanol, 1,2,3,4-tetrahydro-1-naphthol and pentanol) under O_2_ atmosphere, although obtaining conversions below 20% [168]. This anthraquinone **73** was recently used in another reported strategy for the cascade transformation of racemic alcohols to enantiopure amines **74** in moderate to excellent yields, using different ω-transaminases as chiral inductors, sodium phosphate (NaPi), pyridoxal-5-phosphate (PLP) and isopropylamine (IPA), the carbonyl compounds **69** being the intermediates of the reaction (Scheme 19) [169].

9,10-dihydroanthracene (10 mol%) promoted the photo-oxidation of the benzyl alcohol, hexanol, cyclohexanol and furfuryl alcohol to their respective aldehydes and ketones in very good yields, using pure O_2_ and irradiated with a tungsten-bromine lamp [170]. Another observed product from primary alcohols was the corresponding carboxylic acid.

**Figure 21:**
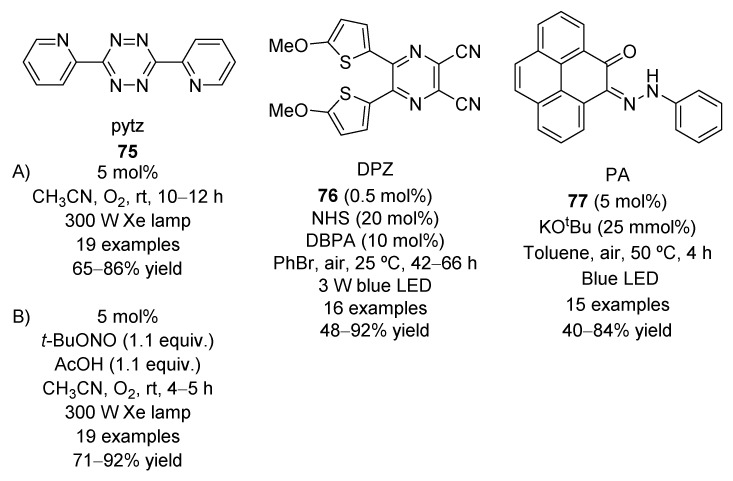
Results obtained in the aerobic photo-oxidation of aromatic and aliphatic alcohols to aldehydes and ketones **69** catalyzed by **75**–**77**.

Apart from the catalysts shown, a group of metal-free nitrogen-containing photocatalysts has been employed to synthesize carbonylic compounds by the aerobic oxidative dehydrogenation of alcohols. This group includes a tetrazine-based catalyst (pytz, **75**) using two methodologies [171], a dicyanopyrazine-derived chromophore (DPZ, **76**) used in cooperation with *N*-hydroxysuccinimide (NHS) and dibenzyl phosphoric acid (DBPA) [172], and an iminoquinone-based catalyst (PA, **77**) [173], enabling all of them to obtain aldehydes and ketones **69** from alcohols in moderate to excellent yields (Figure 21).

**Figure 22:**
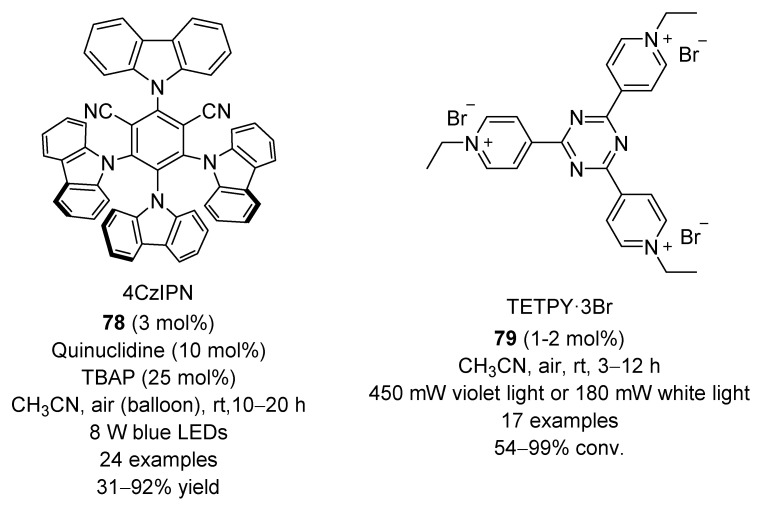
Results obtained in the aerobic photo-oxidation of aromatic and aliphatic alcohols catalyzed by **78** and **79**.

In addition, a carbazole-based promoter (4CzIPN, **78**) with quinuclidine and tetrabutylammonium phosphate (TBAP) as additives [174] and a pyridinium derivative (TETPY·3Br, **79**) [148] were successfully used in the same mentioned transformation (Figure 22). The carbazole photocatalyst **78** has also been employed in a novel strategy of oxidizing alcohols to aldehydes and coupling them in situ with amines to generate the corresponding amides [175].

**Scheme 20:**
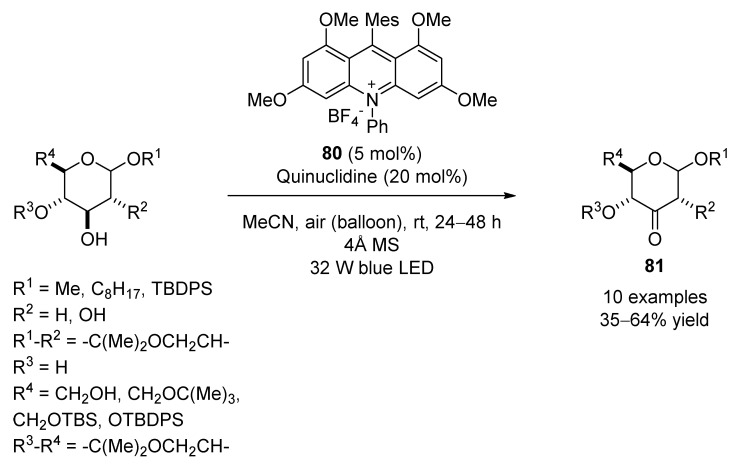
Aerobic photo-oxidation of carbohydrates to ketosugars **81** catalyzed by **80**.

An attractive and selective aerobic oxidative dehydrogenation of carbohydrates achieved after irradiation with visible light under aerobic conditions was recently developed, employing an acridinium salt **80** as catalyst and quinuclidine as HAT mediator, obtaining the corresponding ketosugars **81** in moderate yields (Scheme 20) [176].

**Scheme 21:**
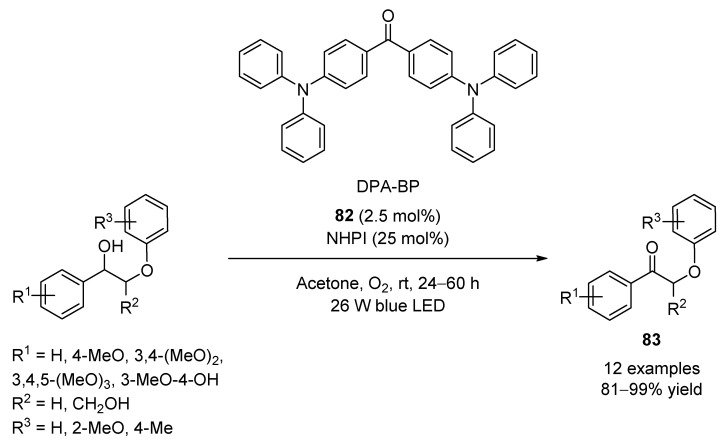
Aerobic photo-oxidation of β-O-4 lignin models catalyzed by **82**.

An approach to the oxidation of different β-O-4 lignin models was developed, employing 4,4′-bis(diphenylamino)benzophenone (DPA-BP, **82**) as photocatalyst and *N*-hydroxyphthalimide (NHPI) as co-catalyst, leading to the corresponding carbonyl compounds **83** in very good yields (Scheme 21) [177]. In the same work, **82** (1.5 mol%) was also used in combination with NHPI (15 mol%) in the aerobic photo-oxidation of 4-methoxyphenethyl alcohol to its acetophenone in 97% yield.

**Scheme 22:**
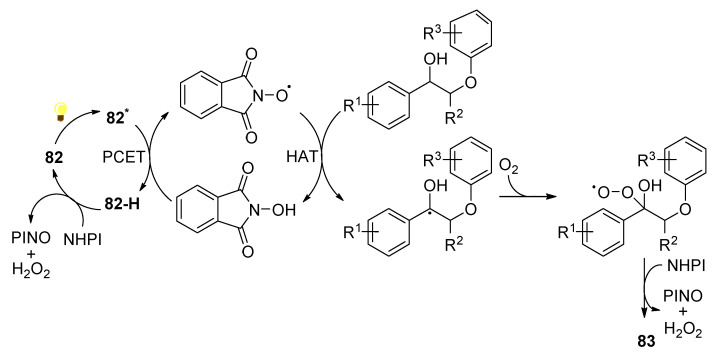
Proposed mechanism for the photo-oxidation of β-O-4 lignin models catalyzed by **82** and NHPI.

The proposed reaction mechanism leading to **83** is shown in Scheme 22. Thus, visible light excites the catalyst **82** to the corresponding excited state 82*. This excited state generates a phthalimide-*N*-oxyl radical (PINO) from NHPI through a proton-coupled electron transfer process (PCET). Then, a hydrogen atom from the alcohol is abstracted by the PINO radical through a HAT process, generating an α–hydroxybenzylic radical and recovering NHPI. This radical reacts with O_2_ to form a peroxy radical and evolves to the final ketone, abstracting a hydrogen atom from NHPI. The same process occurs to recover the catalyst from ketyl **82**-**H** [177].

**Scheme 23:**
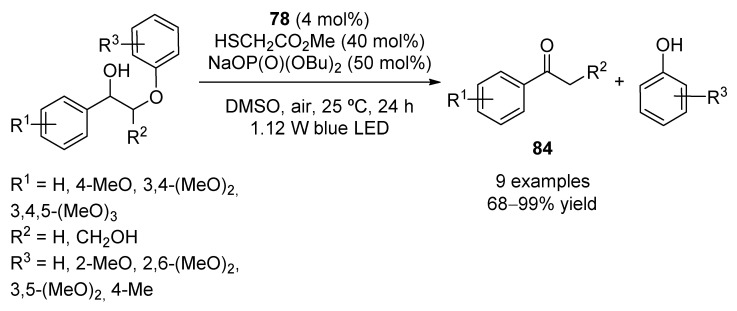
Aerobic photo-oxidative cleavage of lignin model derivatives catalyzed by **78**.

Continuing with lignin models, **78** has been employed as catalyst, in combination with a phosphate and a thiol, in the photo-oxidative fragmentation of lignin model derivatives to obtain the corresponding carbonyl products **84** and the corresponding phenol in moderate to good yields (Scheme 23) [178]. In the same work, the methodology was also applied to diol derivatives presenting several backbones and leaving groups, obtaining good results.

This oxidative cleavage occurs through a similar mechanism to the one shown in Scheme 22 to generate the ketone (in this case the thiol is the co-catalyst). Then, a SET process occurs reducing it to a ketyl radical anion, and the photocatalyst is regenerated. This radical suffers a β C(sp^3^)-O bond cleavage due to its very poor stability, generating a carbonyl radical and an anion from the leaving group. Finally, the radical and the anion are transformed into the corresponding fragmentation products [178].

**Scheme 24:**
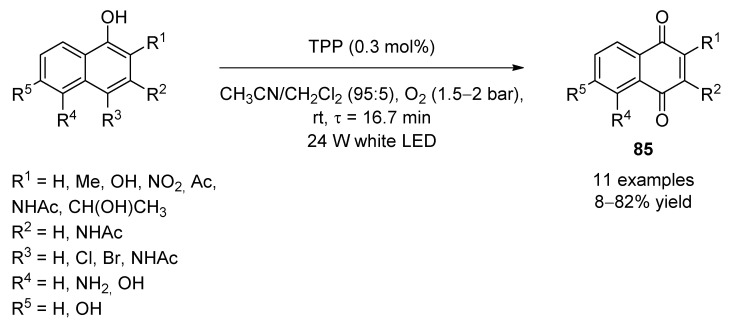
Aerobic oxidative dehydrogenation of naphthols to naphthoquinones **85** catalyzed by TPP.

The aerobic oxidative dehydrogenation of phenols or naphthols to quinones or naphthoquinones, important in some biochemical transformations [179], is another reaction carried out with metal-free catalysts. Thus, TPP was used as photocatalyst in the photo-oxidation of naphthols to naphthoquinones **85** under continuous-flow conditions, obtaining low to good yields (Scheme 24) [180].

**Scheme 25:**
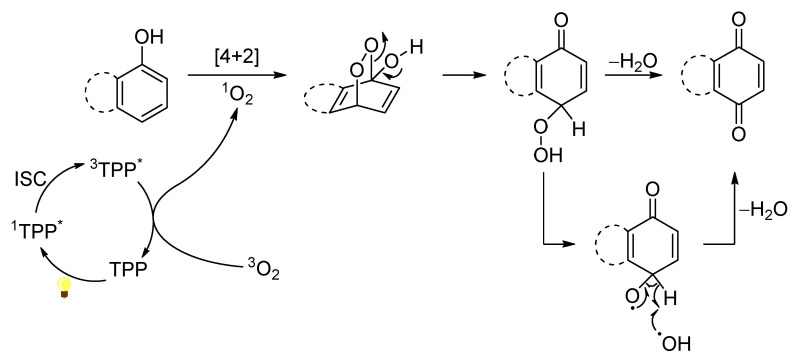
Proposed mechanism of the aerobic oxidative dehydrogenation of naphthols to naphthoquinones **85**.

The proposed mechanism of this reaction is shown in Scheme 25. Firstly, the photocatalyst (TPP) is excited to its singlet excited state (^1^TPP*) by irradiation with visible light. Next, an ISC process is produced to generate the triplet excited state of the catalyst (^3^TPP^•^) from the singlet one. To recover the promoter, triplet oxygen (^3^O_2_) is converted to singlet oxygen (^1^O_2_), which reacts with the starting naphthol by a [4+2] cycloaddition. Then, the formed cycle is opened by an H transposition, and, finally, a molecule of H_2_O is lost to generate the final product.

The same photocatalyst (TPP) was used in the oxidative dehydrogenation of 1,5-dihydroxynaphthalene working under flow conditions, obtaining the corresponding naphthoquinone (Juglone, **85**: R^1^ = R^2^ = R^5^ = H, R^4^ = OH) in very good yield [181]. In addition, it has also been employed in the photo-oxidation of *para*-substituted phenols in a continuous-flow and high-pressure system on a multigram scale using supercritical CO_2_, leading to *para*-hydroperoxy quinones in moderate to excellent yields [182]. This system was also applied to a multistep formation of 1,2,4-trioxanes from *para*-phenol derivatives and alkyl or aromatic aldehydes in very good yields [182].

**Figure 23:**
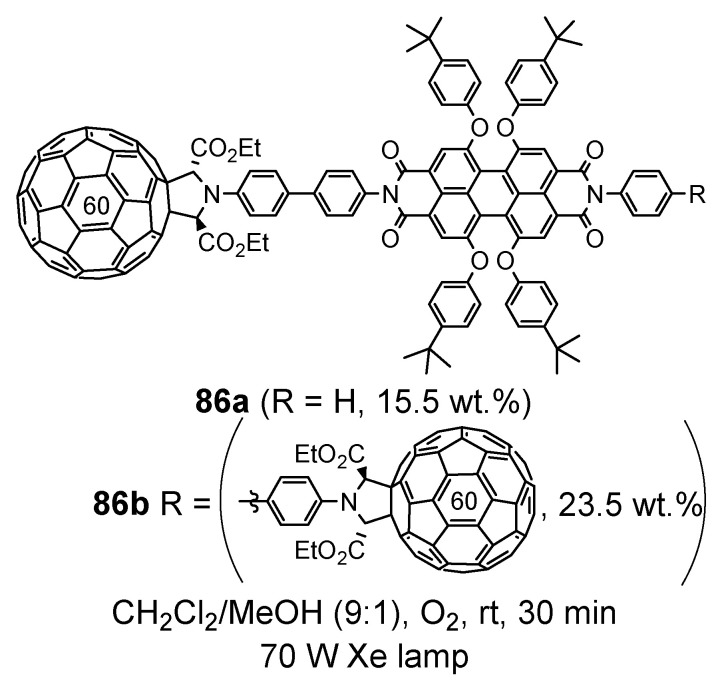
Results obtained in the aerobic oxidative dehydrogenation of 1,5-dihydroxynaphthalene to Juglone (**85**: R^1^ = R^2^ = R^5^ = H, R^4^ = OH) catalyzed by **86**.

Other catalysts used in the photo-oxidation of 1,5-dihydroxynaphthalene to Juglone (**85**: R^1^ = R^2^ = R^5^ = H, R^4^ = OH) were two perylenebisimide-[60]fullerene systems dissolved in CH_2_Cl_2_/MeOH (9:1) (**86a**,**b**) (Figure 23) [183], although only the quantum yield of the transformation was reported.

**Figure 24:**
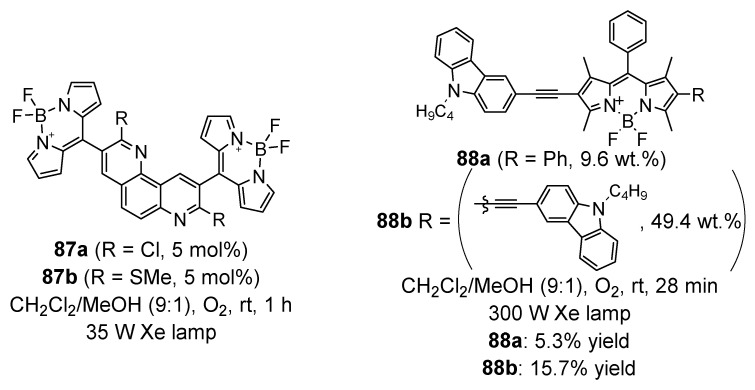
Results obtained in the aerobic oxidative dehydrogenation of 1,5-dihydroxynaphthalene to Juglone (**85**: R^1^ = R^2^ = R^5^ = H, R^4^ = OH) catalyzed by **87** and **88**.

In addition, a mixture of two 1,7-phenanthroline based bis-BODIPYs with Cl- or SMe- substituents (**87a**,**b**) [184] and two BODIPY-carbazole derivatives (**88a**,**b**) [185] photocatalyzed the same oxidation of 1,5-dihydroxynaphthalene to Juglone (**85**: R^1^ = R^2^ = R^5^ = H, R^4^ = OH), only reporting the obtained quantum yield in the first case (Figure 24). Furthermore, methylene blue **1** has been employed in the photo-oxidation of different phenols and naphthols to quinones or naphthoquinones under continuous-flow conditions, obtaining good results [186].

In all these results, it is shown that the oxidative dehydrogenation of C–O bonds to the corresponding carbonyls has been successfully carried out using almost all the recently employed homogeneous photocatalysts, except in the case of some promising flavins. Xanthene-based and nitrogen-containing photocatalysts, such as tetrazines or pyrazines, demonstrated remarkable activity in the mentioned transformation in short reaction times, showing a high capacity to generate ROS. It is worth mentioning that some simple photocatalysts, for instance, **78**, **80** and **82**, as well as TPP, were able to successfully catalyze the photo-oxidation of several alcohols present in sugars, lignin model derivatives and naphthols, resulting in interesting compounds for the pharmacological industry.

### 3.2. Heterogeneous Oxidative Dehydrogenation of C–O Bond

**Figure 25:**
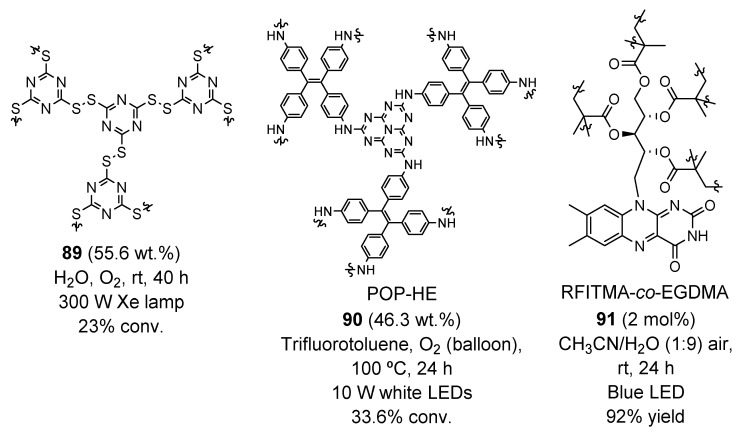
Results obtained in the aerobic photo-oxidation of benzyl alcohol to benzaldehyde catalyzed by **89**–**91**.

The aerobic photo-oxidation of C–O bonds under heterogeneous conditions has been carried out extensively in recent years, using several photocatalysts [7,20,62]. One of the reactions included in this group is the oxidation of primary and secondary alcohols to their carbonyl products. This transformation was performed, for instance, using polymer-based materials as photocatalysts. Thus, the melamine-based polymer **51** promoted the photo-oxidation of benzyl alcohols to the corresponding aldehydes **69** in moderate yields [115]. Other polymers used in this transformation were a trithiocyanuric acid-based material composed of disulfide units **89** (Figure 25) [187], an heptazine-based porous material with ethane-1,1,2,2-tetrayl tetraaniline units (POP-HE, **90**) (Figure 25) [188], a flavin-based mesoporous polymeric network with ethylene glycol dimethacrylate units (RFITMA-*co*-EGDMA, **91**) (Figure 25) [189], and the same hyper-cross-linked material was used in the aerobic oxidative coupling of amines and the dehydrogenation of dibenzylamine [111]. The recyclability of these photocatalysts was successfully carried out by filtration and washing with ethanol and acetone (for **90**) and through a filter-equipped glass reactor (for **91**), while **89** was not reused.

**Figure 26:**
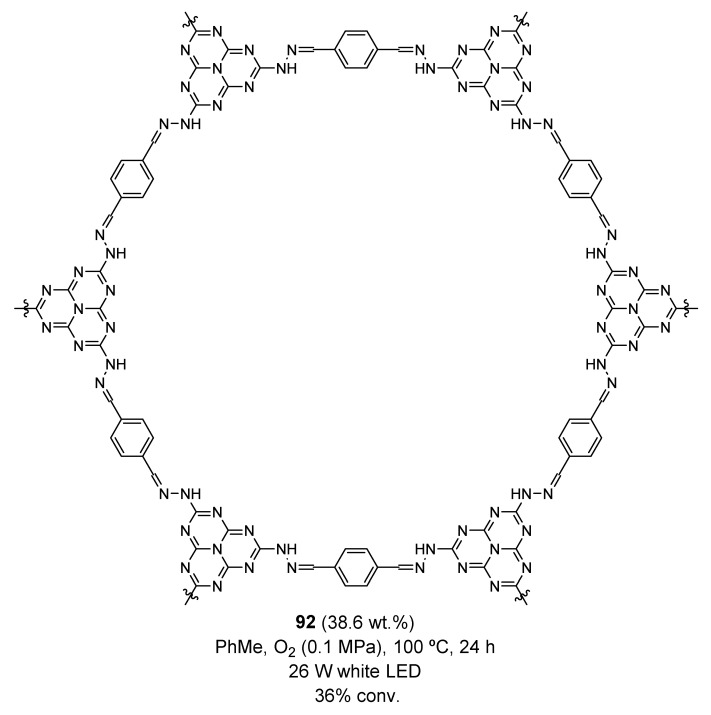
Result obtained in the aerobic photo-oxidation of benzyl alcohol to benzaldehyde catalyzed by **92**.

As in heterogeneous C–N bond oxidative dehydrogenation, carbon nitrides have been used frequently in heterogeneous C–O bond photo-oxidations. In particular, the aerobic oxidation of aliphatic and aromatic alcohols under visible light irradiation has been promoted by several polymeric carbon nitrides. Thus, a melamine-based polymeric promoter [190], a hydrazine-linked heptazine carbon nitride **92** (Figure 26) [191] and a coral-like melamine-based polymeric material [192] were employed in the mentioned transformation (**92** only being employed in the oxidation of the benzyl alcohol to benzaldehyde), obtaining the corresponding aldehydes **69** in moderate to excellent conversions in all cases. The coral-like material was reused by centrifugation and washed with acetonitrile and ethanol, observing similar conversions in all the catalytic cycles. The recycling of the other two photocatalysts was not reported.

Several graphitic carbon nitrides were also employed as photocatalysts in the transformation of alcohols to aldehydes or ketones. Thus, a carbon-nanodot-doped material derived from **33** [193], a few-layered phosphorene nanostructure graphitic material [194], the basic unmodified graphitic carbon nitride **33** [119,195,196] and several **33** modified by thermal, chemical and mechanical processes [197], with N vacancies [198] and doped with P [199], O [200] and S atoms [201], could successfully catalyze the mentioned reaction under visible light irradiation. The photocatalysts were able to be reused after centrifugation or filtration without decreasing their catalytical activity.

**Figure 27:**
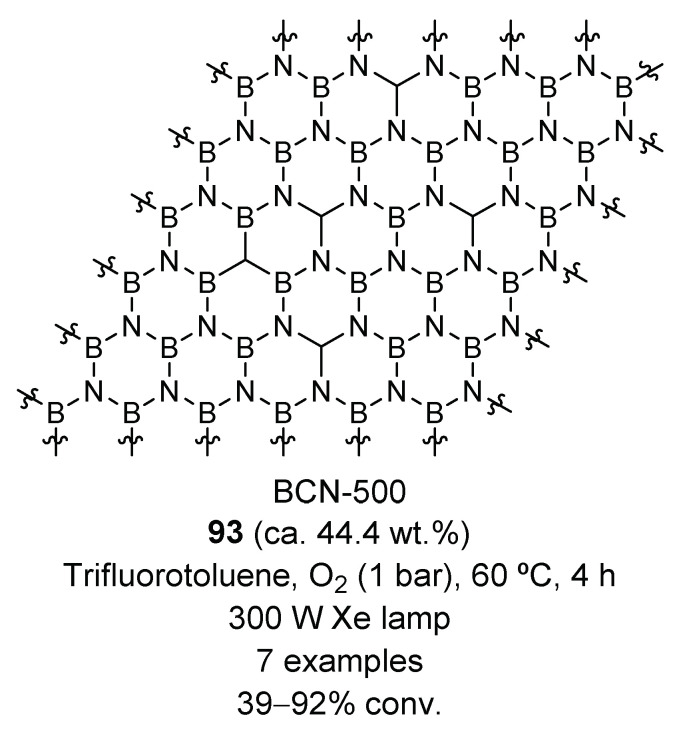
Results obtained in the aerobic photo-oxidation of benzylic alcohols to aldehydes **69** catalyzed by **93**.

Other carbon nitride-based photocatalysts used in the same process were a mesoporous material combined with *N*-hydroxyphthalimide [202], a porous carbon nitride packed in a reactor [123], a boron carbon nitride (BCN-500, **93**) (Figure 27) [203,204], which is a carbon nitride-based photocatalyst applied in energy and environmental areas [205,206,207], and an aerogel composed of carbon nitride nanolayers [208], obtaining the corresponding aldehydes and ketones (only in the first case) from benzyl alcohols in low to excellent conversions. The recyclability of the catalysts was carried out by filtration and washing (for the mesoporous material) and by centrifugation (for **93**), maintaining their activity. The recycling of the other photocatalysts was not reported.

CQDs have also been employed to catalyze the aerobic photo-oxidation of alcohols to carbonyl derivatives **69**. Thus, the use of the oxygen-rich CQDs (O-CQDs, **64**) [144], also successfully employed in the aerobic photo-oxidative coupling of benzylamines (Scheme 2 and Scheme 15), and a polyethylene-based material [209], resulted in the preparation of aldehydes **69** in low or high conversions, respectively. The photocatalysts were recycled by filtration and rinsing in CH_3_CN (for **64**) and by extraction with ethyl acetate (for the polyethylene-based promoter) without any change in their activity.

**Figure 28:**
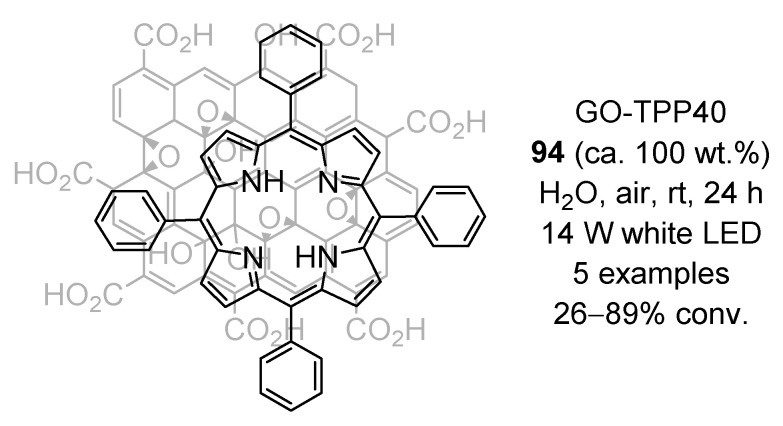
Results obtained in the aerobic photo-oxidation of alcohols to aldehydes **69** catalyzed by **94**.

Similarly, graphene oxide-based promoters have demonstrated good activity in the oxidation of aromatic and aliphatic alcohols leading to the corresponding carbonyl products **69** when irradiated with visible light. In this sense, this reaction was performed well using a graphene oxide/carbon nitride hybrid material [210] and a surface-modified graphene oxide with TPP (GO-TPP40, **94**) (Figure 28) [211], being able to recycle the last one by dispersion in H_2_O and sonication for 1 h with a decrease in its activity in the fourth cycle.

**Figure 29:**
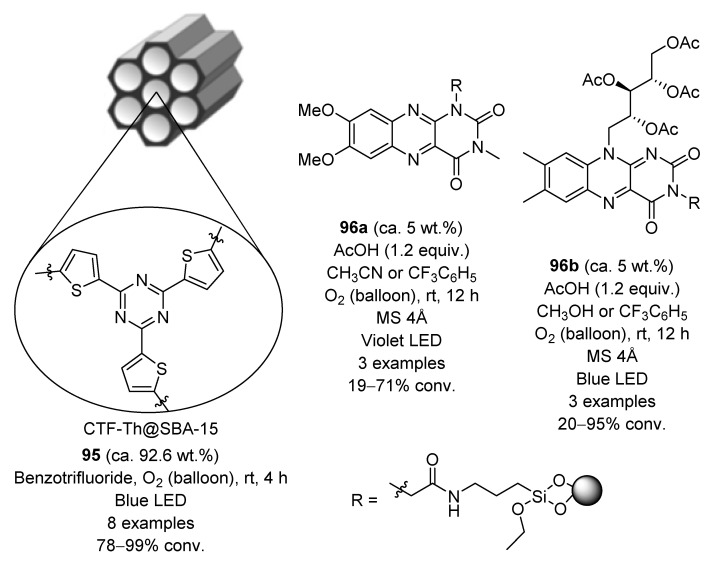
Results obtained in the aerobic photo-oxidation of alcohols to aldehydes or ketones **69** catalyzed by **95** and **96**.

Immobilizing an organic molecule on mesoporous silica is a good strategy for preparing heterogeneous photocatalysts. In this manner, a covalent triazine framework supported on mesoporous silica (CTF-Th@SBA-15, **95**) [212] and two riboflavin derivatives (**96a**,**b**) anchored to mesoporous silica MCM-41 [213] enabled the process of aerobic photo-oxidation of several aromatic alcohols to render aldehydes or ketones **69** in low to excellent conversions (Figure 29). The catalyst **95** was recovered by centrifugation, its activity slightly decreasing along with cycles. In the case of **96**, recycling was not reported.

**Scheme 26:**
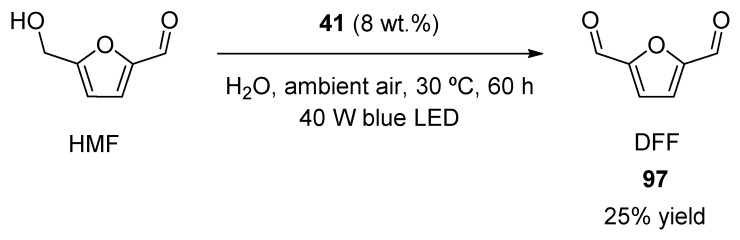
Aerobic oxidative dehydrogenation of HMF to DFF catalyzed by **41**.

The photocatalytic transformation of biomass-derived chemicals has been explored in recent years [214,215,216], and there are some recently reported examples in which metal-free photocatalysts are employed. Starting with polymer-based promoters, the CMP **41** catalyzed the aerobic oxidative dehydrogenation of 5-hydroxymethylfurfural (HMF) to 2,5-diformylfurfural (DFF, **97**) in 25% yield under visible light irradiation (Scheme 26) [217]. The recyclability of **41** was studied, but on a different reaction.

Other metal-free catalysts used in this transformation included a melamine-based polymeric carbon nitride, also employed in the photo-oxidation of two benzyl alcohols to the corresponding aldehydes shown in Scheme 17 [218], the basic graphitic carbon nitride **33** [219,220], a mesoporous carbon nitride [221] and the covalent triazine framework supported on mesoporous silica **95 [222]**. In all cases, moderate conversions to **97** were obtained when irradiated with visible light or using solar light. The photocatalytic materials were recycled by decantation (for the melamine-based polymeric carbon nitride), by decantation and washing with water or centrifugation (for **33**), by washing with water and ethanol (for the mesoporous carbon nitride) and by filtration and washing with water and acetone (for **95**) with no remarkable changes in the results.

The photo-oxidation of the β-O-4 lignin models shown in Scheme 21 has also been carried out under heterogeneous catalysis. Thus, a 3D carbazolic POF generated by coupling carbon radicals from the oxidized carbazole groups of **78** (Figure 22) was employed in this transformation, obtaining **83** in excellent yields [223]. The catalyst was also used in the photo-oxidation of a benzyl secondary alcohol, rendering the corresponding ketone in 99% yield. The recyclability of the material was successfully achieved by centrifugation, observing very similar yields in five catalytic cycles.

**Figure 30:**
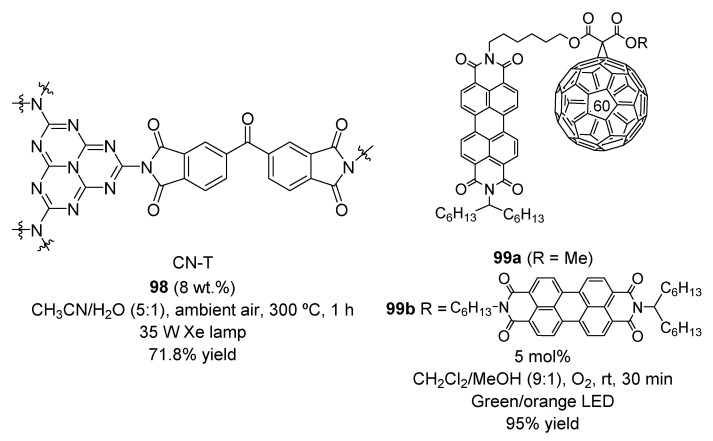
Results of the aerobic photo-oxidation of 1,5-dihydroxynaphthalene to Juglone (**85**: R^1^ = R^2^ = R^5^ = H, R^4^ = OH) catalyzed by **98** and **99**.

Generating quinones and naphthoquinones by oxidative dehydrogenation of phenols and naphthols has been made possible recently by employing different heterogeneous catalysts. Thus, a 3% by weight of methylene blue-dyed polyester fabrics could catalyze the oxidative dehydrogenation of 1,5-dihydroxynaphthalene, obtaining Juglone (**85**, R^1^ = R^2^ = R^5^ = H, R^4^ = OH) in 75% conversion [224]. Other photocatalysts used in the same photo-oxidation of 1,5-dihydroxynaphthalene were a benzoquinone-functionalized carbon nitride (CN-T, **98**) [225] and two perylenebisimide-fullerene dyads (**99a**,**b**) [226], obtaining Juglone (**85**, R^1^ = R^2^ = R^5^ = H, R^4^ = OH) in high yields (Figure 30). Recycling of the methylene blue-dyed polyester fabrics and **99** was not carried out, while **98** was recovered and reused three times, with a slight decrease in its activity at the third catalytic cycle.

**Figure 31:**
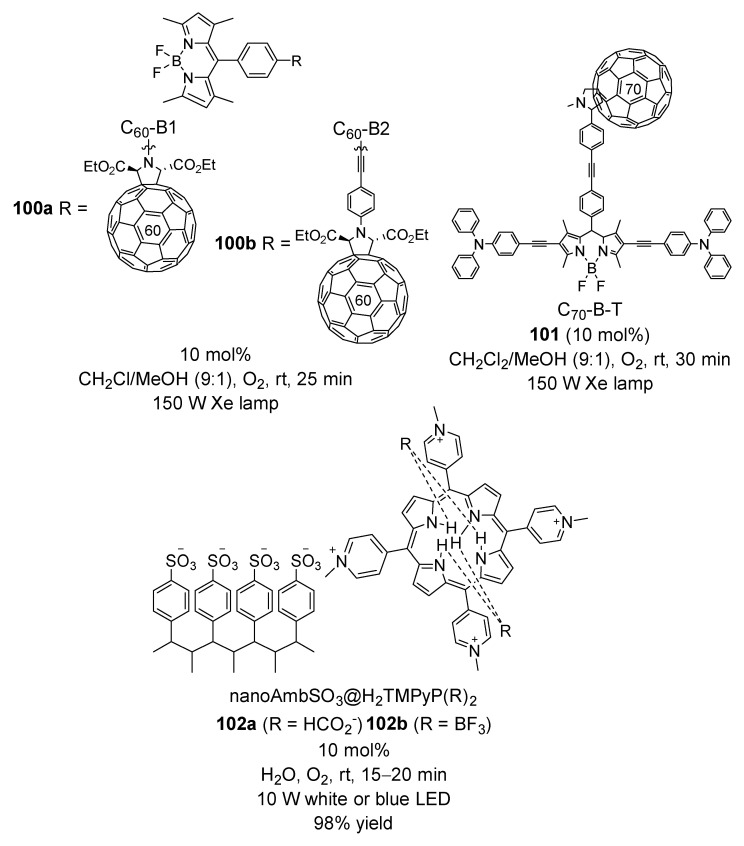
Results of the aerobic photo-oxidation of 1,5-dihydroxynaphthalene to Juglone (**85**: R^1^ = R^2^ = R^5^ = H, R^4^ = OH) catalyzed by **100**–**102**.

In addition, two C_60_-BODIPY photosensitizers (C_60_-B1/C_60_-B2, **100a**,**b**) [227], a C_70_-BODIPY-triphenylamine triad (C_70_-B-T, **101**) [228] and a *meso*-tetrakis(*N*-methylpyridinium-4-yl)porphyrin supported on the sodium salt of Amberlyst 15 nanoparticles and complexed with formic acid (nanoAmbSO_3_@H_2_TYMPyP(HCO_2_H)_2_, **102a**) [229] and BF_3_ (nanoAmbSO_3_@H_2_TYMPyP(BF_3_)_2_, **102b**) [230] were employed in the mentioned transformation, obtaining Juglone (**85**, R^1^ = R^2^ = R^5^ = H, R^4^ = OH) in high yield (only quantum yield was given in the case of using **100** and **101**) (Figure 31). The recyclability of **100** and **101** was not tested, while **102a** and **102b** were recycled but in a different photo-oxidation reaction.

Furthermore, non-complexed **102** in a continuous flow photoreactor [231] and a gigantic porphyrin-based organic cage with 12 units of tetra(4-aminophenyl)porphyrin and 24 moieties of 2-hydroxy-5-octyloxy-1,3-benzenedicarboxaldehyde [232] were used as photocatalysts in the preparation of Juglone (**85**, R^1^ = R^2^ = R^5^ = H, R^4^ = OH) from 1,5-dihydroxynaphthalene in good to excellent yields. However, those catalysts were not recycled.

An oxidative photocatalytic cleavage of the C–C bond of lignin models was recently reported using a mesoporous carbon nitride as photocatalyst, leading to **84** in excellent conversions and moderate yields [233]. In the reaction, the corresponding carboxylic acid from **84** could also be obtained in low to moderate yields. The reusability of the carbon nitride was studied, and the cleavage could be performed again for up to five cycles.

**Scheme 27:**
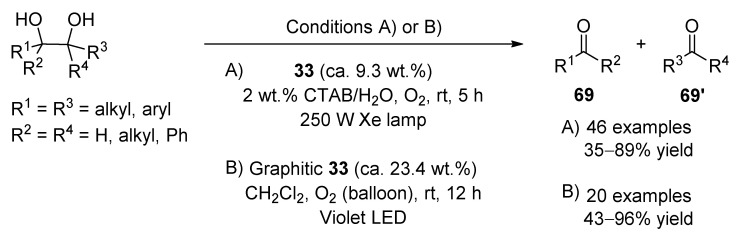
Aerobic photo-oxidative C–C bond cleavage of 1,2-diols catalyzed by **33** or graphitic **33**.

Another recent aerobic oxidative C–C bond cleavage, in this case in vicinal diols, has been reported in two works using the carbon nitride **33** in a micellar medium with cetyltrimethyl ammonium bromide (CTAB) as surfactant [234] and the graphitic form of **33** [235], rendering the corresponding aldehydes or ketones **69** in moderate to good yields and also performing a broad scope of internal and monosubstituted diols (Scheme 27). In the first case, the catalyst was recycled 10 times by extracting the product and adding more starting reagent to the water phase that contained the catalyst. In the case of the graphitic form of **33**, it was recovered by filtration and washing with dichloromethane, being reused for up to five cycles.

**Scheme 28:**
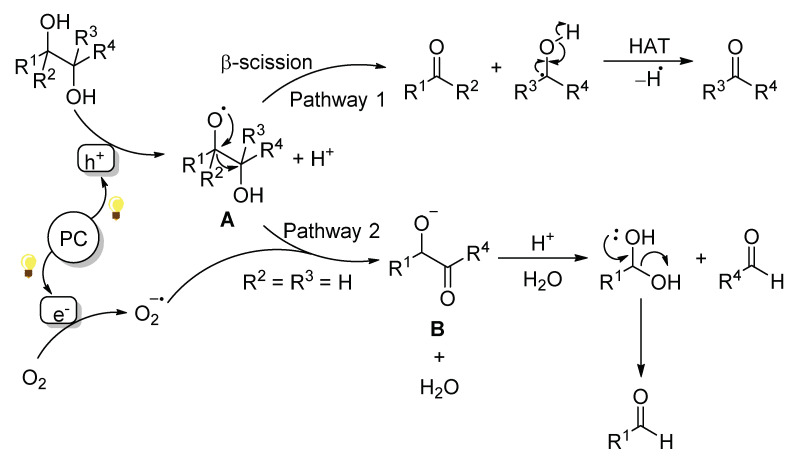
Proposed mechanism for the aerobic photo-oxidative C–C bond cleavage of 1,2-diols.

The proposed mechanism for this aerobic photo-oxidative cleavage of diols is shown in Scheme 28. Firstly, the photocatalyst (PC) is excited, producing electrons (e^−^) and holes (h^+^). Then, one hydroxyl of the 1,2-diols is oxidized by a hole, generating the radical **A** and a proton, while O_2_ is reduced to the superoxide radical by an electron. At this moment, two pathways are possible depending on whether aldehydes or ketones are generated: pathway 1, in which the corresponding aldehyde or ketone and an α-hydroxy radical are generated through a β-scission of the radical **A**, the α-hydroxy radical being transformed subsequently to a second aldehyde or ketone by a HAT process; and pathway 2, in which an intermediate **B** and H_2_O are produced from the reaction between the radical **A** and the superoxide radical, the intermediate **B** being hydrolyzed immediately to generate an aldehyde and a germinal diol that suffers dehydration to render two other aldehydes [235].

Observing the previous examples, it can be deduced that, in terms of reaction yields and conversions, the best photocatalysts for the heterogeneous oxidative dehydrogenation of alcohols to the corresponding carbonyl derivatives were the boron carbon nitride **93**, the graphene oxide/carbon nitride hybrid material **94** and the immobilized ones in silica, while polymer-based photocatalysts promoted transformation in lower yields. In terms of reaction time, the mentioned catalyst types added to BODIPY-based ones promoted different photo-oxidation reactions faster than the other ones. These differences are probably related to different photoabsorptivities and ROS generation abilities. The recyclability of all the photocatalysts was carried out generally simply and conveniently, and all materials were able to be reused for several catalytic cycles, maintaining their activity.

## 4. Conclusions

The oxidative dehydrogenation of C–N and C–O bonds leading to C=N and C=O bonds, catalyzed by metal-free molecules under visible light irradiation and using atmospheric air or pure O_2_ as readily available and cheap oxidant, is a powerful strategy for preparing compounds, such as imines and carbonyl derivatives from amines and alcohols, being also interesting due to the use of environmentally friendly and sustainable reaction conditions. In this review, the recent advances and activities in this field have been shown, a broad variety of metal-free homogeneous and heterogeneous photocatalysts being employed, achieving excellent results. For instance, cheap organic dyes, such as methylene blue or Rose Bengal, are examples of homogeneous promoters used successfully, while polymeric- or carbon nitride-based materials have been used as heterogeneous catalysts among others, showing very good performance and being able to be recovered in most cases without losing their activity, which adds additional value to the methodology. However, some oxidations of biomass-derived chemicals, such as lignin models and 5-hydroxymethylfurfural, are less explored, and the development of more methodologies in this field is necessary. Undoubtedly, the oxidative dehydrogenation of C–N and C–O bonds under these convenient reaction conditions will be explored and improved in the nearest future, particularly in the case of recoverable photocatalysts devoted to their use in flow conditions and applicable to industrial processes.
